# Anti-Biofilm Agents to Overcome *Pseudomonas aeruginosa* Antibiotic Resistance

**DOI:** 10.3390/ph18010092

**Published:** 2025-01-13

**Authors:** Marie Hanot, Elodie Lohou, Pascal Sonnet

**Affiliations:** AGIR, UR 4294, Faculté de Pharmacie, Université de Picardie Jules Verne, 1 Rue des Louvels, 80000 Amiens, France; marie.hanot@u-picardie.fr (M.H.); elodie.lohou@u-picardie.fr (E.L.)

**Keywords:** biofilm, bacterial virulence, *Pseudomonas aeruginosa*, drug design, anti-biofilm agents

## Abstract

*Pseudomonas aeruginosa* is one of world’s most threatening bacteria. In addition to the emerging prevalence of multi-drug resistant (MDR) strains, the bacterium also possesses a wide variety of virulence traits that worsen the course of the infections. Particularly, its ability to form biofilms that protect colonies from antimicrobial agents is a major cause of chronic and hard-to-treat infections in immune-compromised patients. This protective barrier also ensures cell growth on abiotic surfaces and thus enables bacterial survival on medical devices. Hence, as the WHO alerted to the need to develop new treatments, the use of anti-biofilm agents (ABAs) appeared as a promising approach. Given the selection pressure imposed by conventional antibiotics, a new therapeutic strategy has emerged that aims at reducing bacterial virulence without inhibiting cell growth. So-called anti-virulence agents (AVAs) would then restore the efficacy of conventional antibiotics (ATBs) or potentiate the effectiveness of the immune system. The last decade has seen the development of ABAs as AVAs against *P. aeruginosa*. This review aims to highlight the design strategy and critical features of these molecules to pave the way for further discoveries of highly potent compounds.

## 1. Introduction

*Pseudomonas aeruginosa* is a Gram-negative opportunistic bacterium able to survive in many environments and clinical settings. Its large genome (5.5–7 Mb) is composed of a conserved core and an accessory section with high plasticity. Therefore, the bacterium is extremely versatile and can easily adapt to harsh environmental conditions [1,2,3]. *P. aeruginosa* is mostly responsible for pulmonary infections, but is also a major cause of urinary, skin, ear or eye infections, endocarditis and sepsis [4]. In particular, it is one of the leading causes of hospital-acquired infections (notably in intensive care units) and of chronic lung infections in patients suffering from cystic fibrosis (CF) or non-CF bronchiectasis, increasing mortality rates [5,6].

The peculiar pathogenicity of this bacterium is strongly exacerbated by its innate virulence. Firstly, external components of *P. aeruginosa* cells enable the establishment and persistence of infections. Notably, outer membrane lipopolysaccharides (LPSs) protect the pathogen against phagocytosis and oxidative stress, but also promote as covering lectins the adhesion to host tissues. The type IV pili and flagellum allow planktonic cells to move from one infection site to another [7,8]. In addition, the bacterium also secretes virulence factors. Their production is tightly regulated by quorum sensing (QS), the bacterial cell–cell communication network. QS is based on the production, release and perception of small molecules called autoinducers (AIs) that coordinate the expression of specific genes involved in virulence behaviors. In *P. aeruginosa*, three inter-regulated QS systems, *las*, *rhl* and *pqs*, are involved in the secretion of characteristic toxins (pyocyanin pigment, exotoxin A, HCN, exoenzymes ExoA, ExoU, ExoY and ExoS), siderophores (achromobactin, pyoverdines) and diverse proteolytic and lipolytic enzymes (type IV and alkaline proteases, elastases A and B, lipases A and C, phospholipase C) [7,9,10]. Not only can these virulence factors play a significant role in bacterial growth and survival towards immune system response, but they also cause critical damage to host tissues and worsen the clinical course of the infection. More importantly, QS regulates the production of biofilms. These protective structures provide favorable conditions for bacterial persistence, antibiotic (ATB) tolerance and recurrence of the infection. Biofilms are clusters of bacterial communities embedded in an extracellular matrix mainly composed of polysaccharides, nucleic acids, proteins, lipids and water [11]. This barrier offers protection against harsh external conditions (temperature, pH, physical stress) and against the immune system. The proximity between different bacterial species favors the transmission of genetic material including resistance-related genes [12]. These properties enable micro-organism dissemination in many environments including medical devices (catheters, implants, prosthetic valves or joints, endotracheal tubes) and in vivo tissues. Antibiotic resistance is also enhanced by this microenvironment in which sessile cells exhibit higher genotypic and phenotypic tolerance than planktonic forms. The MICs of antibiotics against bacterial biofilms are 10 to 1000 times higher than against planktonic cells [13,14]. The matrix prevents the penetration of polar antibiotics such as tobramycin while the low oxygen and nutrient levels in deeper layers of the biofilm alter the effect of aminoglycosides, quinolones or beta-lactams by slowing down bacterial metabolism, resulting in low protein, DNA or peptidoglycan synthesis [15,16]. These dormant strains are able to tolerate high antibiotic concentrations and eventually repopulate the biofilm, leading to chronic infections [17].

*P. aeruginosa* is one of the deadliest bacteria. In 2019, it was responsible for more than 500,000 deaths, which can be explained by its particular virulence, as well as the prevalence of multi-drug-resistant (MDR) strains [18]. Clinical isolates show reduced susceptibility to a wide variety of antibiotic classes including beta-lactams, fluoroquinolones, polymyxins and aminoglycosides. Notably, over the past years, resistances to carbapenems, ceftazidime and the piperacillin–tazobactam combination have increased [19]. The propensity of *P. aeruginosa* to become resistant to the whole arsenal of antibiotics is due to numerous factors [20]. First, the complex lipopolysaccharidic diderm membrane barrier of the pathogen naturally decreases permeability to antimicrobials, and the associated efflux pumps expel broad varieties of toxic molecules including antibiotics [21,22]. *P. aeruginosa* also easily develops resistances through mutation of intrinsic genes or acquisition of genetic material by horizontal gene transfer [23,24]. In particular, phenotypic modifications of the pharmacological targets can spontaneously appear upon external stimuli. To date, loss of the OprD porin outer membrane channel, overexpression of efflux pumps and production of AmpC (or extended-spectrum) beta-lactamases are the most commonly reported resistance phenotypes in *P. aeruginosa* MDR isolates [25]. This phenomenon is increased by the misusage of ATBs that triggers a selection pressure favorable to the multiplication of resistant strains. Accordingly, the WHO has alerted to the necessity to design new therapeutical strategies to fight this threatening pathogen. A new anti-virulence approach has recently emerged that aims at quenching bacterial virulence without affecting cell growth. Such anti-virulence agents (AVAs) could restore the efficacy of conventional ATBs in combination therapy. In monotherapy, they could potentiate the pathogen’s eradication by the immune system.

The development of anti-biofilm agents (ABAs) seems like an interesting strategy to alleviate the burden caused by *P. aeruginosa* infections. ABAs are specific AVAs that diminish virulence by targeting the biofilm. ABAs and, more generally, AVAs differ from ATBs by their lack of effect on bacterial cell growth. In this bibliographic review, the most promising synthetic ABAs designed as anti-virulence molecules were presented. Hence, these ABAs are able to hinder the formation or the dispersion of biofilm without eradicating sessile microcolonies. They can act as (i) c-di-GMP pathway inhibitors (slowing down the production of biofilm or activating its dispersion), (ii) QS inhibitors (QSIs) (diminishing the production of matrix components and AIs as well as toxin secretion) or (iii) lectin inhibitors (impairing the biofilm structure). Some conventional ATBs or antimicrobial peptides have also been studied for their anti-biofilm properties at concentrations that do not affect bacterial growth, as has the nanovectorization biotechnological approach allowing an extracellular matrix permeabilization. We aim to highlight the critical features that enable molecular key interactions with targets to pave the way for the rational development of new ABAs against *P. aeruginosa*.

## 2. The Biofilm Life Cycle

**Figure 1:**
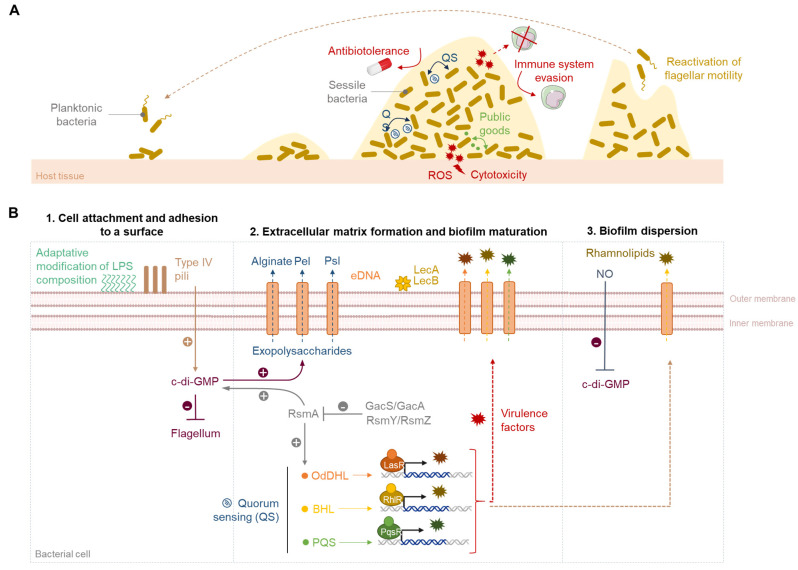
The biofilm life cycle (**A**) and related signaling pathways (**B**).

Biofilms are elaborate structures of bacterial communities attached to a surface and supported by a self-produced matrix mainly constituted of polysaccharides, nucleic acids, proteins, lipids and water. Their development can be divided in five stages: (i) the reversible attachment of bacterial cells to a surface, (ii) their irreversible adhesion, (iii) the initial formation of the extracellular matrix and proliferation of microcolonies, (iv) the biofilm maturation and finally (v) the biofilm dispersion and bacterial dissemination (Figure 1).

### 2.1. Cell Attachment

**Figure 2:**
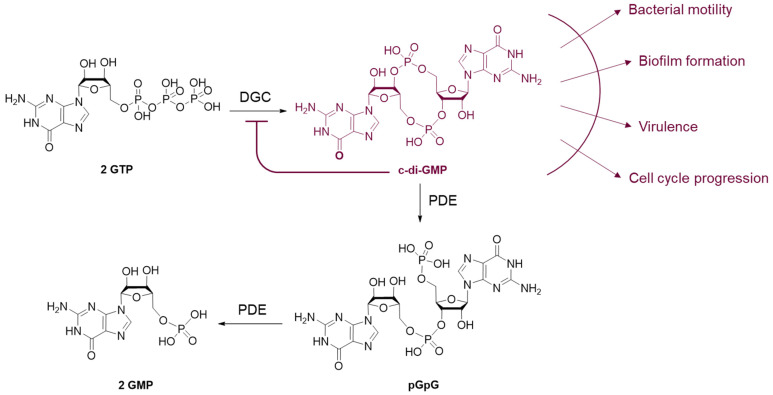
Synthesis and enzymatic degradation of c-di-GMP and its role in cell function. Diguanylate cyclases (DGCs) synthesize c-di-GMP from two molecules of guanosine triphosphate (GTP). Phosphodiesterases (PDEs) hydrolyze c-di-GMP into pGpG, then two molecules of guanosine monophosphate (GMP). c-di-GMP controls bacterial motility, biofilm formation, cell cycle progression and virulence by binding to different receptor/effector proteins. The c-di-GMP molecule appears in purple.

The initial attachment of *P. aeruginosa* to a biotic or abiotic surface relies on its physicochemical properties (mechanical roughness, hydrophobicity) and other different environmental factors such as pH, temperature or nutrient levels. The flagellum plays a major role in the establishment of the first reversible van der Waals interactions by overcoming the repulsive forces between the cell and the surface [26,27]. Then, upon recognition of a surface, type-IV pili (T4P) activate the production of bis-(3′,5′)-cyclic dimeric guanosine monophosphate (c-di-GMP) that is a secondary messenger regulating biofilm production in several bacterial species [28]. According to environmental stimuli, diguanylate cyclases (DGCs) synthesize c-di-GMP or phosphodiesterases (PDEs) catalyze its degradation (Figure 2). The production of c-di-GMP is regulated by the two-component system GAC (Global activator of Antibiotic and Cyanide synthesis) composed of the regulator GacA and the transmembrane kinase GacS. GacA activation mediated by the phosphorylation of GacS stimulates the production of small RNAs RsmZ and RsmY. Those bind to the RsmA protein to inhibit its transcriptional activity and lead to the biosynthesis of DGC. The c-di-GMP signaling pathway is also in charge of flagellum expression. Increasing levels of c-di-GMP brake flagellum-based motility and allow the cell transition from a motile to a sessile state [29]. This phenotypic change includes modifications in the lipopolysaccharide (LPS) composition, promoting cell–cell aggregation and cell surface adhesion. The LPS is composed of a polysaccharidic region called O-antigen, an oligosaccharidic core and lipid A inserted in the outer membrane. *P. aeruginosa* produces two types of O-side chains. The most common type termed “A-band” is electroneutral at physiological pH, whereas the “B-band” O-side chain contains electronegative sites [30]. The bacterium can thus tune the cell surface hydrophobicity (A-band) or hydrophilicity (B-band) in the function of the physicochemical properties of the external medium [31]. Finally, high c-di-GMP levels trigger the production of extracellular matrix components, mainly exopolysaccharides which will maintain the biofilm’s irreversible adhesion to the surface.

### 2.2. Irreversible Adhesion and Biofilm Formation

The main intracellular signaling pathway governing biofilm formation and maturation mechanisms is quorum sensing (QS) [26]. QS is a bacterial communication system which regulates gene expression in proportion to cell density. When the quorum is reached, small extracellular molecules called autoinducers (AIs) are released and bind to their receptors in a neighboring cell to activate the transcription of virulence genes. In *P. aeruginosa*, three QS circuits named *las*, *rhl* and *pqs* are described. The *las* and *rhl* systems can be found in several pathogens. They produce and recognize acyl-homoserine lactone-like AI: the *N*-(3-oxododecanoyl)-L-homoserine lactone (3-oxo-C_12_-HSL) odDHL and the *N*-butanoyl-L-homoserine lactone (C_4_-HSL) BHL, that activate their transcription factors LasR and RhlR, respectively. A third specific *pqs* system produces and detects the 2-heptyl-3-hydroxy-4(1*H*)-quinolone PQS (*Pseudomonas* quinolone signal). This AI activates its transcription factor PqsR.

The extracellular aqueous polymeric matrix is composed of proteins, rhamnolipids, extracellular DNA (eDNA) and exopolysaccharides named Pel, Psl and alginate [32]. Psl and Pel maintain structural integrity and are necessary for the cell adhesion to biotic or abiotic surfaces. Their production is stimulated by c-di-GMP. The alginate is essentially secreted by mucoid strains in the lungs of CF patients in response to stressful conditions (nutrients deprivation, antibiotics, slower cell growth, high osmotic pressure). In addition to preserving matrix structural cohesion, the alginate helps evade the immune system by resisting phagocytosis and macrophage-mediated free radical damage, but also diminishes antibiotic diffusion [33,34,35]. eDNA, which is excreted following eukaryotic cell lysis induced by pyocyanin, is mainly a cell–cell interconnecting component. It contributes to the mushroom-like structure of the biofilm, can serve as a nutrient source and appears as a proinflammatory molecule by activating neutrophils. It can also reduce antibiotic penetration and promote horizontal gene transfer, and it takes part in the coordination of cell movements during twitching motility, thus contributing to biofilm expansion [33,36]. In the early stages of biofilm formation, soluble lectins A and B (LecA and LecB), aggregating proteins implemented at the outer membrane surface, strengthen the biofilm stability by binding to matrix and membrane saccharides from other cells [37]. At this stage, rhamnolipids stimulate the LPS composition adaptation and the swarming motility. After irreversible adhesion and production of the extracellular matrix, bacteria grow in microcolonies and proliferate in the biofilm.

### 2.3. Biofilm Maturation

During the maturation phase, QS and c-di-GMP regulate the production of matrix components so that the biofilm reaches a stable tridimensional structure. In this advantageous niche, bacteria are protected against biocides, the host immune system, antibiotics or other deleterious environmental conditions. The rhamnolipids create channels so that nutrients can circulate through the biofilm. The species diversity in the biomass favors horizontal gene transfer, which is a way for micro-organisms to acquire fitness genes. Hence, various phenotypes emerge, creating small colony variants that contribute to tissue damage and resistance. They notably possess more pili and produce more polysaccharides and c-di-GMP, which is why they better adhere to abiotic surfaces [38]. Interestingly, the advantageous microenvironment created by the extracellular matrix enables the establishment of QS-mediated bacterial interactions, in the form of collaboration or competition between various species. Bacteria collaborate via the exchange and share of public goods, secreted enzymes or chelators that help the digestion of nutrients. However, the presence of cheaters (i.e., bacteria that benefit from public goods without producing any) can be a threat to the biofilm community. The different bacterial species can also directly or indirectly compete with other pathogens. They can steal nutrients such as iron from their rivals, ensure the degradation of their signaling molecules, enhance biofilm dispersion or manipulate the host immune system to eradicate them. Furthermore, bacteria also secrete antimicrobials such as bacteriocines or respiratory chain inhibitors. For example, *P. aeruginosa* and *Staphylococcus aureus* often form mixed biofilms in infected lungs of CF patients [39]. *P. aeruginosa* synthetizes then the 4-hydroxy-2-heptylquinoline-*N*-oxide (HQNO) that inhibits *S. aureus* growth. However, it also protects *S. aureus* from antibiotics such as tobramycin or vancomycin, because of cell metabolism reduction. Interestingly, the increased alginate production in mucoid strains of *P. aeruginosa* reduces the secretion of anti-staphylococcal molecules and enables the cohabitation of the two species within the biofilm [35]. These interactions also result in the overproduction of virulence factors that cause severe damage to host tissues and stimulate the inflammatory reaction [40].

### 2.4. Biofilm Dispersion and Bacterial Dissemination

The dispersion of the biofilm is an essential part of its life cycle. Several factors such as a lack of nutrients or oxygen, strong competition or an outgrown population can induce a partial or complete dispersal. Released bacteria quickly revert to a planktonic phenotype and colonize another surface that is richer in nutrients and more supportive for growth [41]. It is noteworthy that the biofilm can also undergo a mechanical erosion induced by environmental physical forces such as flow velocity. Those cells are then more likely to retain a sessile state. Spontaneous dispersal is mainly triggered by the production and detection of different signal molecules. For instance, nitrous oxide (NO) and glutamate diminish the c-di-GMP levels, thus increasing flagellar expression and promoting bacterial motility. On top of that, QS stimulates the secretion of rhamnolipid biosurfactants which create cavities in the extracellular matrix to facilitate the detachment of microcolonies [42]. This dispersion promotes the dissemination of the infection, in particular on abiotic devices in which bacterial biofilms rapidly develop. Ventilation-associated pneumonia therefore significantly increases mortality among hospitalized patients. In such a situation, the removal of the device is the most effective way to eliminate the source of the infection.

Considering the increased bacterial pathogenicity and resistance in biofilm-related infections, the development of anti-biofilm agents appears to be a relevant therapeutic strategy.

## 3. Anti-Biofilm Agents to Tackle *P. aeruginosa*

Biofilms act as protective niches against immune defenses and antibiotics. Therefore, they play a major role in antimicrobial resistance and the establishment of chronic diseases. The steady increase in knowledge about biofilms is guiding the development of ABAs of interest. Several in vitro models have been described as well as various ways to evaluate the anti-biofilm activity of compounds. Firstly, the biofilm can be grown in static conditions and formed in microplates or small surfaces like membranes or coupons. Secondly, dynamic conditions in which biofilm settles in flow cells can be implemented. These independent chambers allow biofilms to grow in the presence of a shear force and a continuous nutrient supply.

The quantification of biomass is then carried out following one of the following methods: (i) crystal violet (CV) staining and optical density (OD) measurement, (ii) biofilm visualization by fluorescence microscopy, (iii) adherent cell isolation followed by OD measurement or counting of colony forming units (CFUs)/mL. The biofilm is grown in microplates in the presence of the tested compounds, in conditions that favor its formation (supplementation of the culture medium with essential cations). After planktonic cell elimination and rinsing of the wells, the biofilm is stained with a CV solution, then dissolved and quantified by UV/Visible spectrometry (OD_595_). The biofilm is grown in microplates or on surface units in the presence of the tested compounds and different fluorescent probes which allow the evaluation of the production of its components. This monitoring can also be realized by measuring (i) the fluorescence from a modified strain that secretes a fluorophore like the green fluorescent protein (GFP) or (ii) the luminescence from a bioluminescent strain expressing for instance the luciferase gene. The biofilm is grown in microplates or surface units in the presence of the tested compound. Adherent cells are removed, diluted in an appropriated medium and inoculated on agar plates. The counting of the colonies allows the estimation of bacterial concentration in CFU/mL reflecting the initial biomass [43].

**Figure 3:**
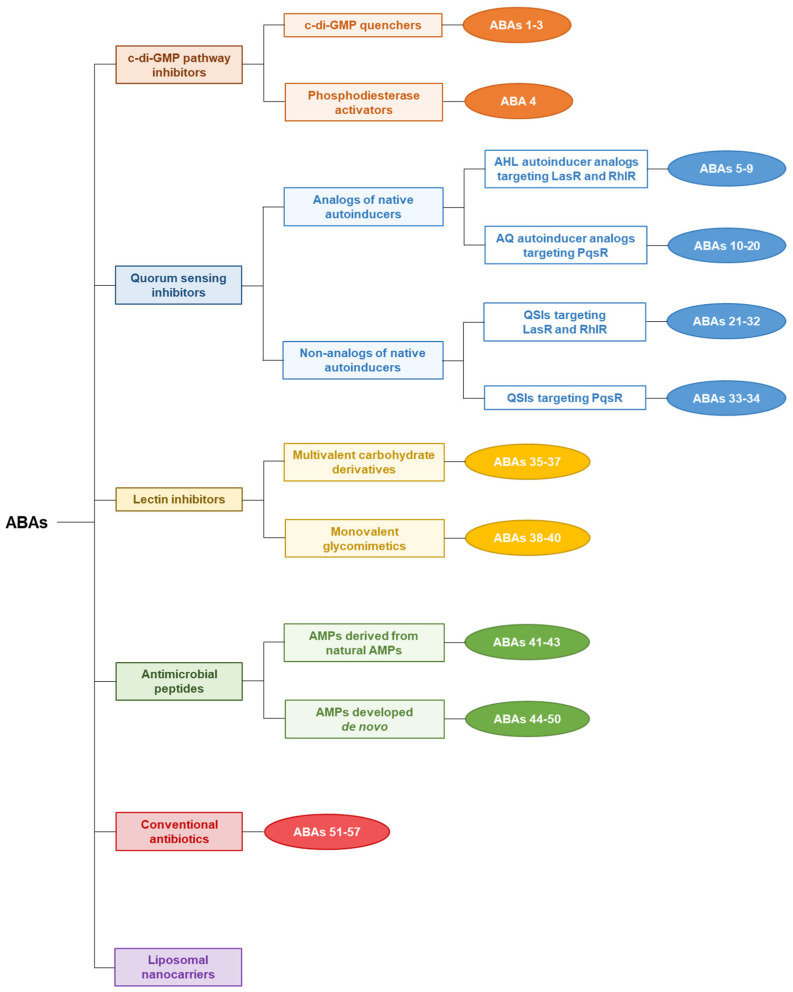
Classification of ABAs described in this review.

It should be noted that the efficacy of ABAs described in the literature is difficult to compare due to the diversity of experimental procedures. In this review, the promising ABAs will be classified according to their target involved in the signaling pathways or the scaffolding of biofilms, and then according to the chemical structure of their pharmacophore (Figure 3). Particular attention will be paid to key interactions between compounds and their pharmacological target studied by molecular docking, in particular those which appear crucial for the anti-biofilm activity.

### 3.1. Relevant Anti-Virulence Agent Review

#### 3.1.1. c-di-GMP Signaling Pathway Inhibitors

The c-di-GMP molecule plays a significant role in the regulation of biofilm formation and dispersion, making it a very attractive target for the development of anti-biofilm agents [38].

**Figure 4:**
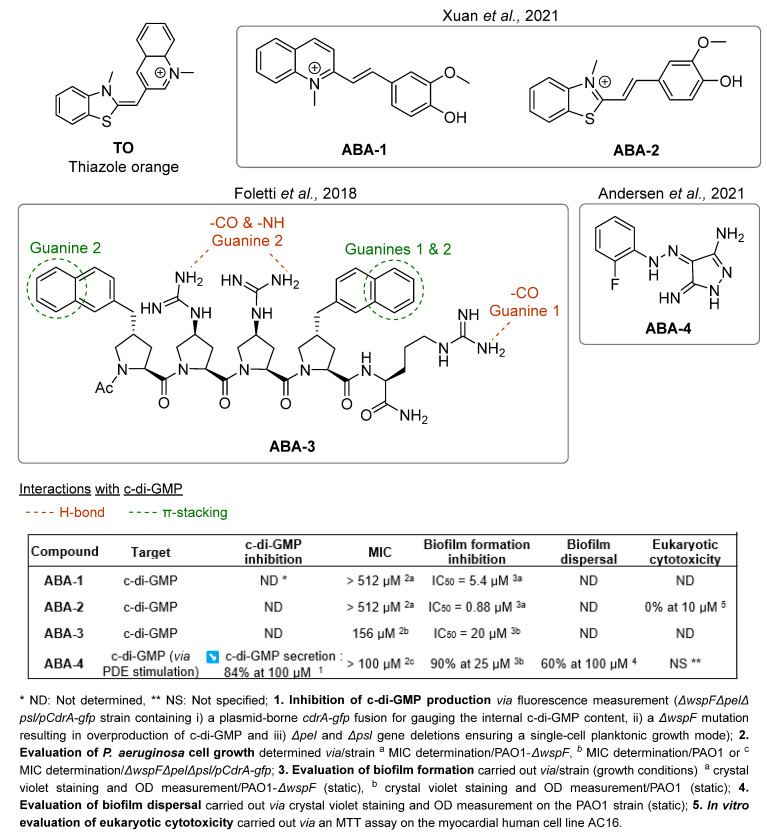
Key interactions with the target and anti-biofilm activities of c-di-GMP signaling pathway inhibitors [44,45,46].

The peculiar structure of c-di-GMP enables the establishment of strong interactions with planar aromatic molecules, thus forming highly stable complexes. Interestingly, these aggregates can quench the activity of c-di-GMP. Hence, several studies have focused on the development of small intercalating molecules to bind c-di-GMP and reduce biofilm production in *P. aeruginosa*. Thiazole orange (TO) has been extensively studied as a potent c-di-GMP inactivator via the formation of tetramolecular complexes. However, TO is highly cytotoxic because of its ability to intercalate into DNA. Xuan et al. have designed two series of cationic quinolines and benzothiazoles inspired by TO to obtain non-toxic compounds active against *P. aeruginosa* (Figure 4) [44]. Structure–activity relationship (SAR) studies revealed the natural importance of the substituents on the phenyl ring. Particularly, the presence of hydrogen bond donors or acceptors enhanced the activity whereas electron-withdrawing groups in *ortho*-position decreased or suppressed it. The derivatives **ABA-1** and **ABA-2** exhibited promising anti-biofilm activities on the PAO1-*ΔwspF* mutant (overexpressing c-di-GMP) with respective inhibitory concentration 50% (IC_50_) values of 5.4 and 0.88 µM. The authors highlighted the ability of **ABA-2** to diminish exopolysaccharide production through the reduced expression of the *PelA* gene. **ABA-2** did not induce cytotoxicity on human myocardial cells below 10 µM, nor did it intercalate into DNA, contrary to TO. It is noteworthy that the nature of the interactions between these compounds and c-di-GMP (i.e., π-π stacking and hydrogen bonds) determines the stabilization of the complex. This was demonstrated by the study of Foletti et al. on short-chain proline-based peptides like **ABA-3** [45]. Those structures are not only biocompatible but also easily modifiable, which allows specific tunings to refine their binding to c-di-GMP. The functionalized proline residues were chosen for their capacity to provide conformational stability to the peptides and interact specifically with c-di-GMP. Particularly, the cationic guanidinoprolines (Gups) of **ABA-3** could establish hydrogen bonds with guanines and phosphate units of c-di-GMP, while the naphtyl-prolines (Naps) would mostly interact via π-π stacking. The pentapeptide **ABA-3** bearing two Nap residues at its *C*- and *N*-terminus extremities forms a very stable homodimer sandwich-like complex with c-di-GMP molecules (*K*_d_ = 1.7 µM). **ABA-3** inhibits biofilm formation with an IC_50_ of 20 µM. Yet, cytotoxicity on eukaryotic cells could be a major drawback for these structures and has unfortunately not been reported.

Intercalating agents being relatively cytotoxic, another strategy has emerged that aims at stimulating c-di-GMP degradation via the activation of phosphodiesterase enzymes. Andersen et al. [46] brought forward **ABA-4**, a pyrazole-derived molecule able to reduce the levels of intracellular c-di-GMP by 84% at 100 µM in a wild-type strain of *P. aeruginosa*, without inhibiting cell growth (Figure 4). It also inhibits 90% of biofilm formation at 25 µM and induces 60% dispersal of an established biofilm at 100 µM. A first preclinical study on a mouse model has proved the in vivo efficacy of **ABA-4**, which eliminated 90% of adherent cells on bacteria-coated implants in the peritoneal cavity, at 1 mg/kg. Further studies indicated that the compound’s activity is dependent on the expression of the *bifA* PDE gene, which is highly conserved among the pseudomonal strains, but not in other bacterial species. As a result, this molecule is a narrow-spectrum anti-biofilm agent. In an original way, the authors highlighted the real potential of enzyme stimulation in the research of novel therapies. In addition, they pointed out the interest in the development of new anti-biofilm agents of the key structural pyrazole scaffold, often present in anti-cancer, anti-inflammatory or antimicrobial compounds.

The development of c-di-GMP pathway inhibitors appears to be a very promising strategy to inhibit biofilm formation and/or to induce the dispersal of a pre-established matrix. However, the use of planar aromatic compounds able to complex with c-di-GMP is limited by their cytotoxicity inherent to their intercalating properties. The design of the highly potent molecule **ABA-4** inducing c-di-GMP degradation via PDE stimulation has still brought forward new perspectives for future optimizations.

#### 3.1.2. Quorum Sensing Inhibitors

Considering the major regulating role played by QS in the development of biofilms, the design of quorum sensing inhibitors (QSIs) as anti-biofilm agents appears as a promising and sustainable solution.

**Figure 5:**
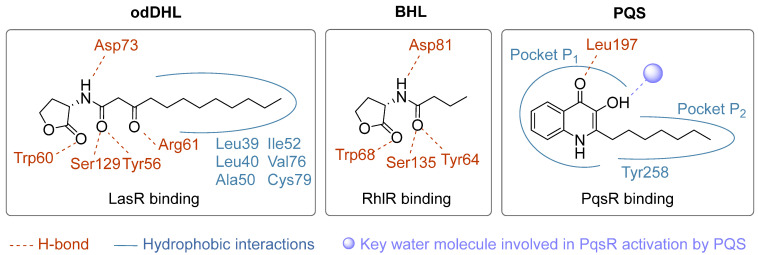
Essential interactions between natural autoinducers odDHL, BHL and PQS and their corresponding receptors LasR, RhlR and PqsR.

Most of these classes of ABAs target one of the three transcription factors, LasR, RhlR or PqsR, from the *las*, *rhl* or *pqs* systems, respectively. Therefore, the comparison of their binding modes with those of acyl-homoserine lactone (AHL) or 2-alkyl-4(1*H*)-quinolone (AQ) autoinducers appears relevant. For this study, some protein/ligand complexes are characterized and available in the Protein Data Bank (PDB) (Figure 5). The signaling molecules odDHL and BHL bind to their respective receptors LasR and RhlR in a similar manner: (i) the carbonyl group of the lactone moiety establishes a hydrogen bond with a tryptophan residue (Trp60 for LasR and Trp68 for RhlR), (ii) the secondary amine forms a hydrogen bond with an aspartic acid residue (Asp73 for LasR and Asp81 for RhlR) while (iii) the 1-oxo group from the acyl chain is involved in two H-bonds with serine and tyrosine residues (Ser129/Tyr56 for LasR and Ser135/Tyr64 for RhlR). The additional carbonyl group in odDHL enables the establishment of a key H-bond with Arg61. Finally, the alkyl chain is surrounded by hydrophobic residues. Interestingly, while the alkyl chain of odDHL appears to be buried in the hydrophobic pocket of the LasR ligand-binding domain, the shorter chain of BHL is not as tightly surrounded by residues in the corresponding RhlR hydrophobic pocket. This could favor the RhlR binding of molecules other than the native ligand. The ligand-binding domain of PqsR is composed of two pockets, P_1_ and P_2_. The deep planar-shaped pocket P_1_ allows the quinolone moiety of the autoinducer PQS to fit into a unique orientation contacting with the Leu207, Leu208, Ile236, Ile149, Ala168 and Phe221 residues, while the cigar-like long pocket P_2_ hosts the heptyl chain thanks to a key interaction with Tyr258 [47,48,49]. Moreover, Shandil et al. describe the establishment of an H-bond between the quinolone carbonyl group and Leu197 (NH) [50]. Finally, a water molecule acts in the apo protein form as an H-bond donor with Leu208 and Arg209 and as an H-bond acceptor towards Gln194, thus blocking the conformation of the autoinduction site. The interaction of the 3-OH group of PQS with this water molecule could destabilize this binding network and be responsible for the activation of the receptor.

In the following sections, the most promising anti-biofilm QSIs will be described and divided in two main categories: analogs and non-analogs of native AIs implicated in the *las*, *rhl* and *pqs* systems.

##### Analogs of Native Autoinducers

(1) AHL autoinducer analogs targeting LasR and RhlR

**Figure 6:**
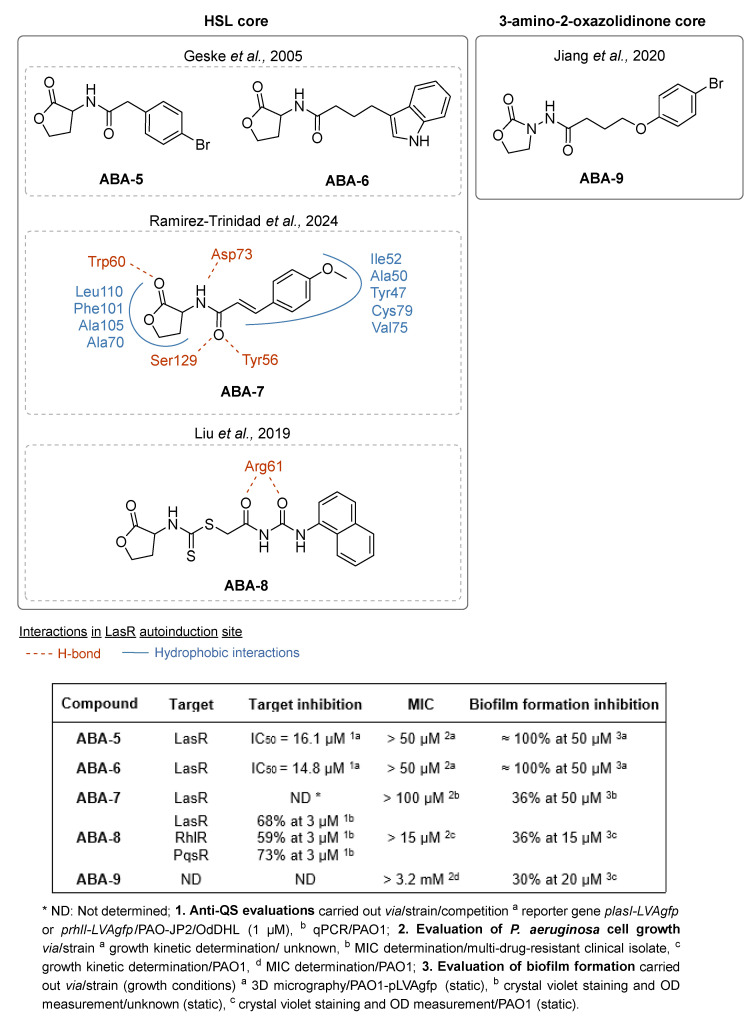
Key interactions with the target(s), anti-QS and anti-biofilm activities of AHL autoinducer analog QSIs [51,52,53,54].

A first strategy for the development of odDHL and BHL analogs has been the modification of the long acyl chain by the introduction of diverse aromatic rings. The development by Geske et al. of novel AHL analogs by solid phase synthesis gave them easy access to a wide variety of derivatives [51]. Notably, the introduction of bromophenyl and indole groups led to **ABA-5** and **ABA-6**, which inhibit LasR with IC_50_ of 16.1 and 14.8 µM, respectively. Thanks to their anti-LasR activity, these two compounds inhibit 100% of biofilm formation at 50 µM (Figure 6). In 2024, the *N*-acyl chain of homoserine lactone (HSL) was substituted by Ramirez-Trinidad et al. by a cinnamyl group [52]. Interestingly, the compound **ABA-7** bearing this conjugated carbonyl system and methoxy electron-donating group in *para* position of the phenyl ring reduced biofilm formation by 36% at 100 µM. Molecular docking studies revealed the formation of key hydrogen bonds between the HSL nucleus and Trp60, Asp73, Ser129 and Tyr56 residues in the autoinduction site of LasR, similar to those formed by the native autoinducer odDHL.

Other modifications of the acyl chain of native AI consisted in introducing various spacers between the HSL core and an aromatic fragment, as described by Liu et al. with the analog **ABA-8** [53]. The presence of a dithiocarbamate group enables the molecule to form two additional H-bonds between the phenylurea and the key residue Arg61 (Figure 6). In fact, **ABA-8**, which is the most active compound of the series, is the only molecule able to establish these essential interactions for potent anti-LasR activity. With a potent efficacy on all three QS systems (68%, 59% and 73% inhibition of LasR, RhlR and PqsR at 3 µM, respectively), **ABA-8** inhibits biofilm formation by 36% at 15 µM.

Finally, Jiang et al. reported the replacement of the HSL core with a 3-amino-2-oxazolidinone nucleus, well-known for its antimicrobial properties. [54] Pharmacomodulations on this scaffold brought out the compound **ABA-9** bearing a three-carbon acyl chain ended by a phenoxy group substituted by a bromine in its *para* position (Figure 6). **ABA-9** revealed a good anti-biofilm activity with 30% inhibition of biofilm formation at 20 µM.

No cytotoxicity data have been reported for those molecules.

(2) AQ autoinducer analogs targeting PqsR

**Figure 7:**
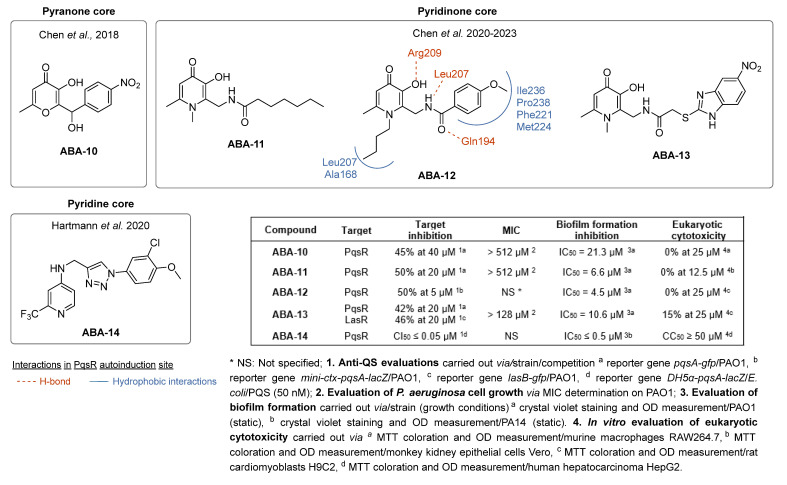
Key interactions with the target(s), anti-QS and anti-biofilm activities of AQ autoinducer analog QSIs bearing a pyranone, pyridinone or pyridine core [55,56,57,58,59].

The design of AQ analogs is mainly based on the use of quinolone bioisosters (Figure 7). Notably, Chen et al. designed four ABAs based on pyranone and pyridinone patterns. First, the compound **ABA-10** was isolated from a series of 3-hydroxy-6-methyl-4*H*-pyran-4-ones substituted by a benzyl group in position 2 [55]. In fact, the *para* substitution with a nitro group of this aromatic ring gave the best anti-biofilm activity with an IC_50_ of 21.3 µM against the PAO1 strain. Interestingly, **ABA-10** showed no cytotoxicity below 50 µM in an MTT assay on murine macrophage RAW264.7 cells. Then, the authors demonstrated the relevance of the pyridinone scaffold, often found in iron chelators, for combining biofilm inhibition and iron trapping [56]. Their study explored various side alkyl chain lengths and different linkers between aromatic rings. The best compound, **ABA-11**, potently reduced biofilm formation (IC_50_ of 6.6 µM) with an innovative mode of action. Indeed, the inhibition of QS systems is associated with a vectorization allowed via recognition by the pyoverdine receptor Fpva. The cytotoxicity of this siderophore analog appeared very weak on Vero monkey kidney cells (15% cell viability inhibition at 25 µM). From 2022, this research team combined the amide spacer of **ABA-11** with the suitably substituted aromatic ring of **ABA-10** [57,58]. This work brought out the compounds **ABA-12**, which possesses a benzene core with a *para* substitution by a methoxy group, and **ABA-13,** carrying a 5-nitro-benzimidazole nucleus inspired by the biaromatic molecule **M64** (**ABA-33**) described as a reference ABA. **ABA-12** showed the best activity by repressing biofilm formation with an IC_50_ of 4.5 µM thanks to its selective antagonism towards PqsR (50% inhibition at 5 µM). **ABA-12** established key interactions in the autoinduction site of PqsR such as H-bonds with Arg209, Leu207 and Gln194 residues. Moreover, this compound displayed no eucaryotic cytotoxicity on rat cardiomyoblast cells H9C2 below 25 µM. This novel *pqs*-selective QSI also revealed interesting anti-pyocyanin activity (IC_50_ = 8,6 µM). In vivo, this pseudomonal anti-virulence efficacy resulted in a drastic increase in the survival rate of infected zebrafish from 10 µM. However, its MIC has not been specified. Its analog **ABA-13** inhibits biofilm formation with an IC_50_ of 10.6 µM thanks to antagonist activity towards both PqsR and LasR without affecting growth. This molecule is slightly more cytotoxic than **ABA-12** (15% Vero cell viability inhibition at 25 µM). Interestingly, a combination treatment with ciprofloxacine and **ABA-12** at 10 µM or **ABA-13** at 20 µM diminished the MIC of the antibiotic on the reference strain PAO1 and some clinical multi-drug-resistant isolates. **ABA-12** also proved to have synergistic activity with tobramycin and colistin E.

Finally, other PQS analogs bearing a 4-aminopyridine core have been patented by Hartmann et al. [59]. In particular, the compound **ABA-14** inhibits PqsR in a competitive manner with a very low IC_50_ inferior or equal to 0.05 µM. As an inverse agonist of PqsR, **ABA-14** reduces the biofilm formation in the PA14 strain with an IC_50_ inferior or equal to 0.5 µM and does not demonstrate significant cytotoxicity on eukaryotic cells (cytotoxic concentration 50% (CC_50_) ≥ 50 µM on human hepatocarcinoma cell line HepG2).

**Figure 8:**
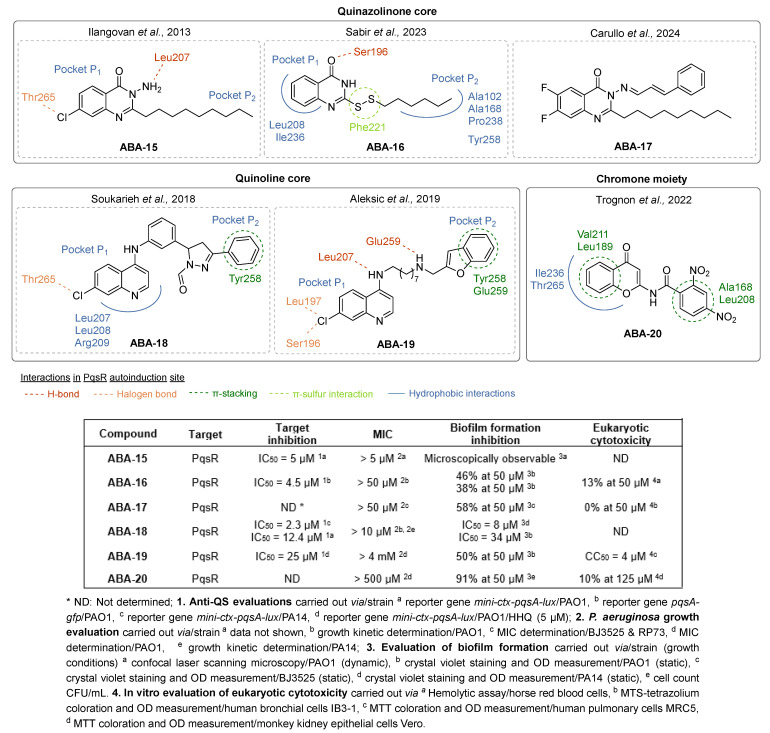
Key interactions with the target, anti-QS and anti-biofilm activities of AQ autoinducer analog QSIs bearing a quinazolinone, quinoline or chromone core [47,60,61,62,63,64,65].

Ilangovan et al. identified the quinazolinone scaffold as a potent pharmacophore for PqsR inhibition, associated with good anti-biofilm properties [47]. Their study notably highlighted the similar positioning of the hit compound **ABA-15** in the autoinduction site of PqsR as PQS establishing hydrophobic interactions (Figure 8). The quinazolinone core occupies the pocket P_1_ just as the quinolone nucleus of the AI and the alkyl chain extends in the pocket P_2_. Interestingly, the 7-Cl atom of **AAB-15** forms a halogen bond with Thr265 in a vacant subpocket. The 3-NH_2_ function appears essential for the competitive inhibition since it establishes an H-bond with Leu207, a key residue in the activation of PqsR by its native AI. The anti-pqsR activity of **AAB-15** correlates with good anti-virulence efficacy including a microscopically observable reduction in biofilm formation and a decrease in pyocyanin secretion of 50% at 50 µM. Although the eukaryotic cytotoxicity of this AVA has not been determined, this pharmacophore has served as a starting point for extensive pharmacomodulations over the years. In 2023, Sabir et al. added a disulfide bridge to the alkyl chain in position 2, a pattern which plays a significant role in the QSI efficacy of several compounds in the literature [60]. The molecule **ABA-16** displayed the best anti-PqsR activity with an IC_50_ of 4.5 µM, thanks to a similar location in the two hydrophobic pockets as PQS, making key interactions with Ser196, Leu208 and Tyr258. The disulfide linkage enables the establishment of an additional binding with the Phe221 aromatic ring. Accordingly, **ABA-16** exhibited a potent inhibition of biofilm formation in the PAO1 strain by 46% at 50 µM. The authors also described its synergistic activity with ciprofloxacin, without relevant eukaryotic cytotoxicity at anti-virulence concentrations. In combination with 50 µM of **AAB-15**, the MIC of the antibiotic was reduced by half. Then, Carullo et al. investigated the effect of quinazolin-4(3*H*)-one derivatives on the virulence of two clinical strains of *P. aeruginosa* (BJ5325 and RP73), representative of acute and chronic lung infections, respectively [61]. The compound **ABA-17** bearing two fluor atoms in positions 6 and 7 of the quinazolinone as well as a cinnamic imine in position 3 showed the most interesting efficacy against biofilm formation with a 58% reduction at 50 µM on the BJ3525 strain. It revealed no cytotoxicity on bronchial cells IB3-1 at this concentration. However, **ABA-17** did not decrease the secretion of pyocyanin as much as other compounds of the series, which is why it was not studied further.

Soukarieh et al. designed 7-Cl-4-aminoquinolines as isosteres of the 7-Cl-quinazolinone pharmacophore described by Ilangovan et al. They replaced the hydrophobic alkyl chain at the C2 position of the quinazolinone by a succession of aromatic rings at the N4 position of the 4-aminoquinoline leading to **AAB-18** (Figure 8) [62]. As well as for its quinazolinone analogs, a molecular docking study revealed that the aminoquinoline resides in the planar pocket P_1_ while the aromatic tail extends in the long pocket P_2_. The chlorine in position 7 of the core forms, like for **ABA-15**, a halogen bond with Thr265. Accordingly, **ABA-18** displayed very potent biofilm formation inhibitory properties with IC_50_ of 8 and 34 µM on the PA14 and PAO1 strains, respectively. It also sensitized at these concentrations preformed biofilms from both strains to a tobramycin treatment (100 µg/mL), leading to a significant eradication of the colonies. Aleksic et al. also described the 7-chloro-4-aminoquinoline analog **ABA-19** for which a benzofurane-substituted aminoalkyl chain was introduced at the N4 position [63,64]. The presence of the two secondary amines allows the formation of H-bonds with Leu207 and Glu259 while a π-stacking settles between the benzofurane moieties Tyr258 and Glu259. Unlike compounds **ABA-16** and **ABA-18**, the 7-Cl atom forms halogen bonds with Leu197 and Ser196. Thus, **ABA-19** inhibited PAO1 biofilm formation by 50% at 50 µM. However, this result has to be moderated because the compound reduced bacterial growth by 25% at this concentration. It also showed strong cytotoxicity towards pulmonary fibroblasts (MRC5) with a CC_50_ of 4 µM.

In 2022, Trognon et al. synthesized PQS analogs by replacing the quinolone with a chromone core, known for its diverse therapeutical applications (anti-inflammatory, anti-tumor, anti-diabetic, vasodilator or antibacterial properties) and which can easily be functionalized [65]. Guided by docking enthalpies of various studied ligands in the autoinduction site of PqsR, the authors replaced the heptyl chain at the C2 position by an amide spacer followed by an aromatic ring (Figure 8). Two series were then developed: a direct-and a retro-chromone carboxamide series, meaning that the chromone could be either linked to the ketone or amine part of the amide. The retro-chromone carboxamide series exhibited the best biofilm formation inhibitors, with no cytotoxicity towards eukaryotic cells. Among those, the 2,4-dinitro-*N*-(4-oxo-4*H*-chromen-2-yl) benzamide **ABA-20** revealed 91% anti-biofilm activity at 50 µM. A molecular docking study pointed out the formation of π-stacking and hydrophobic interactions with key residues such as Leu208, Thr265 or Ile236. **ABA-20** was therefore selected as a hit compound for further optimizations.

The development of AI analogs as QSIs seems an interesting way to achieve competitive inhibition of the target. Most of them show very potent anti-biofilm properties. Yet, these compounds are not always specific to only one transcription factor. There seems to be a fine line between the design of an antagonist and that of an agonist.

##### Non-Analogs of Native Autoinducers

(1) QSIs targeting LasR or RhlR

**Figure 9:**
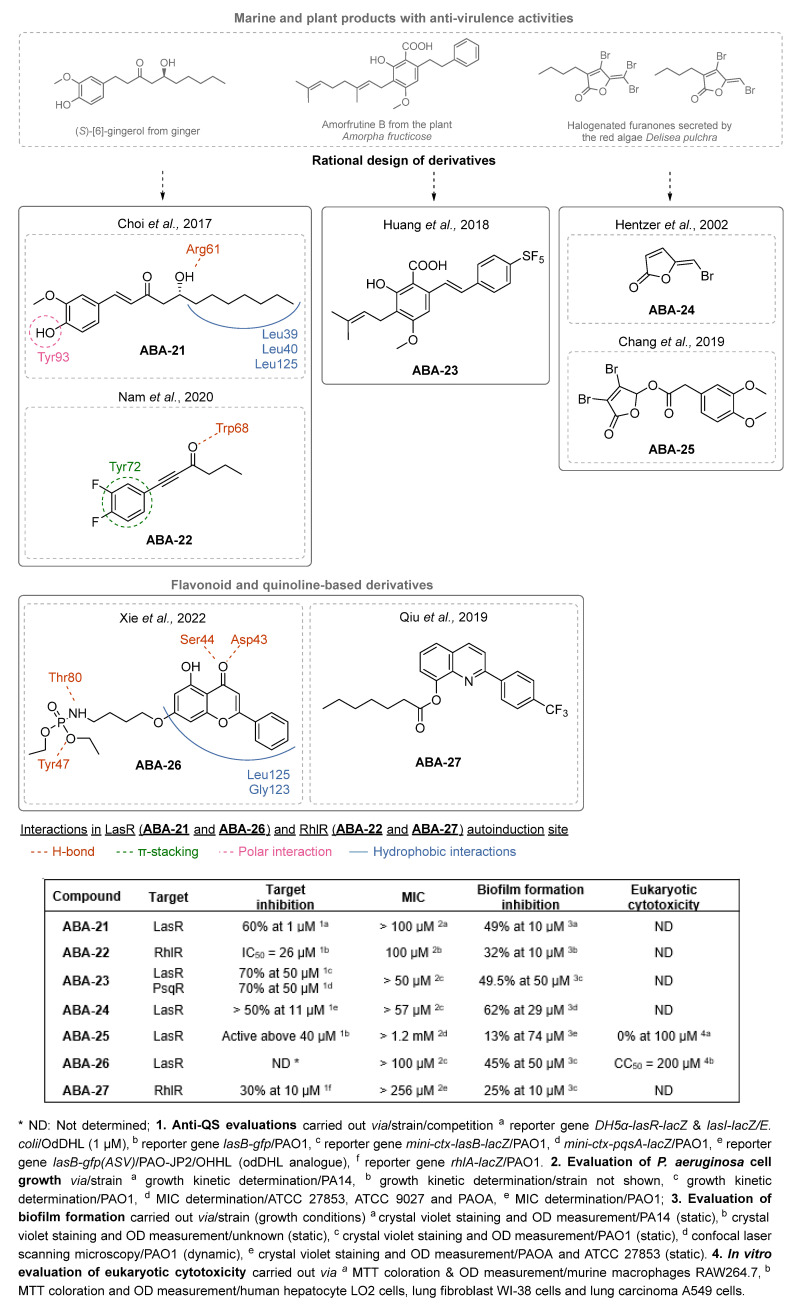
Key interactions with the target, anti-QS and anti-biofilm activities of QSIs inspired from natural compounds targeting LasR or RhlR [66,67,68,69,70,71,72,73].

Some natural products possess anti-virulence activity, including anti-biofilm activities. Hence, they constitute a basis for interest in the synthesis of ABAs whose biological and physicochemical properties can easily be optimized. Choi et al. pointed out the ability of (*S*)-[6]-gingerol, the main component of ginger, to potently decrease *P. aeruginosa* virulence by competing with odDHL for LasR binding [66]. Based on these results, the team performed SAR studies starting from different derivatives to develop ABAs. Molecular modeling screening indicated the importance of the 3′-hydroxyl-4′-methoxyphenyl moiety of (*S*)-[6]-gingerol for LasR binding. The presence of a hydrogen or halogen bond acceptor at the 4-position (-F or -OH) was especially crucial for the activity. Regarding the alkyl chain, the affinity for LasR increased with higher lengths, i.e., 8-gingerol vs. 6-gingerol, which maximize van der Waals interactions with lipophilic amino acid residues (Figure 9). Interestingly, removal of the hydroxyl group on the alkyl tail decreased the anti-biofilm activity, while forming the (*R*)-enantiomer increased the efficacy. Finally, the introduction of a double bond between the phenyl moiety and the carbonyl group confirmed the importance of restricted rotational flexibility for the affinity to LasR and the anti-biofilm properties. The (*R*)-[8]-gingerol derivative **ABA-21,** as a LasR competitive antagonist, exhibited 49% anti-biofilm activity at 10 µM on the PA14 strain. Then, Nam et al. designed new (*S*)-[6]-gingerol analogs with shorter alkyl chains in an attempt to mimic BHL and bind competitively to RhlR [67]. They developed two series of 3′,4′-difluorophenyl derivatives bearing various alkynyl ketones or alcohols. The *n*-butyryl compound **ABA-22** displayed the best activity with 32% inhibition of biofilm formation at 10 µM and even more than 72% in dynamic culture conditions. The exopolysaccharide and protein composition of the matrix are then decreased by 39 and 72%, respectively. Interestingly, the restricted flexibility between the phenyl and carbonyl groups significantly increased the RhlR affinity (IC_50_ = 26 µM) while the removal of the β-hydroxyl group of the natural analog appeared inconsequential. Molecular docking studies revealed a π-stacking between the phenyl ring and Tyr72 as well as an H-bond between the carbonyl and Trp68. Remarkably, longer alkyl chains weaken these interactions, which is correlated with a loss of the antagonist activity towards RhlR. **ABA-22** also reduces rhamnolipid production by 42% at 10 µM, which could slow down bacterial colonization and biofilm formation. This molecule has emerged as one of the only relevant specific inhibitors of RhlR described in the literature. Huang et al. were interested in the development of cajaninstilbene acid derivatives, especially starting from amorfrutin B, which possesses potent anti-biofilm properties. SAR studies led to the compound **ABA-23**, which inactivates LasR and PqsR and diminishes by 50% the biofilm formation at 50 µM [68,69]. In 2002, Hentzer et al. synthetized halogenated furanones inspired by analogs produced by the marine macroalga *Delisea pulchra* and which specifically interfere with AHL-regulated bacterial communication processes [70]. Among those new derivatives, **ABA-24** inhibited the biofilm formation of the PAO-JP2 strain by 62% after 7 days of exposure at 29 µM in dynamic culture conditions. More recently, Chang et al. designed other halogenated furanone derivatives, leading to the discovery of **ABA-25**, which inhibits 31% of biofilm formation at 74 µM [71]. This hit compound displays low eukaryotic cytotoxicity and interesting ADME properties. It is noteworthy that several other furanone analogs have been described as QSIs. Although lacking significant anti-biofilm activity, they possess very promising anti-virulence properties resulting from their good affinity towards LasR. Finally, the quinoline and flavonoid natural scaffolds have also triggered interest due to their antimicrobial properties, which led to many derivatives potentially able to interfere with AHL-associated communication systems. However, high concentrations of these compounds were necessary to successfully inhibit QS. Yet, Xie et al. described the screening of a flavonoid library that enabled the discovery of LasR inhibitor **ABA-26** via a reporter gene assay [72]. This product reduces biofilm formation by 45% at 50 µM, with very low cytotoxicity towards various human cell lines. A molecular docking study of **ABA-26** revealed hydrophobic interactions between the flavonoid core and the residues Leu125 and Gly123 as well as H-bonds between its carbonyl group and Asp43 and Ser44 (NH). Furthermore, the study highlighted the importance of the phosphoramidate function as it could induce a conformational change of Tyr47 next to the LasR autoinduction site, thus destabilizing the activated transcription factor and inducing the dissociation of LasR from DNA. Indeed, **ABA-26** does not seem to compete with the native ligand in the autoinduction domain, which could be in favor of an allosteric inhibitory mechanism. Although quinoline derivatives are known for their anti-virulence activity as AQ autoinducer analogs, the compounds developed by Qiu et al. showed a specificity towards RhlR [73]. The fluorine-substituted phenyl group of **ABA-27** and the alkyl chain of its substituent in position 8 of the quinoline proved to be essential for anti-biofilm activity. In particular, the latter is known for the establishment of van der Waals interactions in the hydrophobic pockets of the BHL receptor. **ABA-27** inhibited 25% of biofilm formation at 10 µM and reduced 25% of rhamnolipid production.

**Figure 10:**
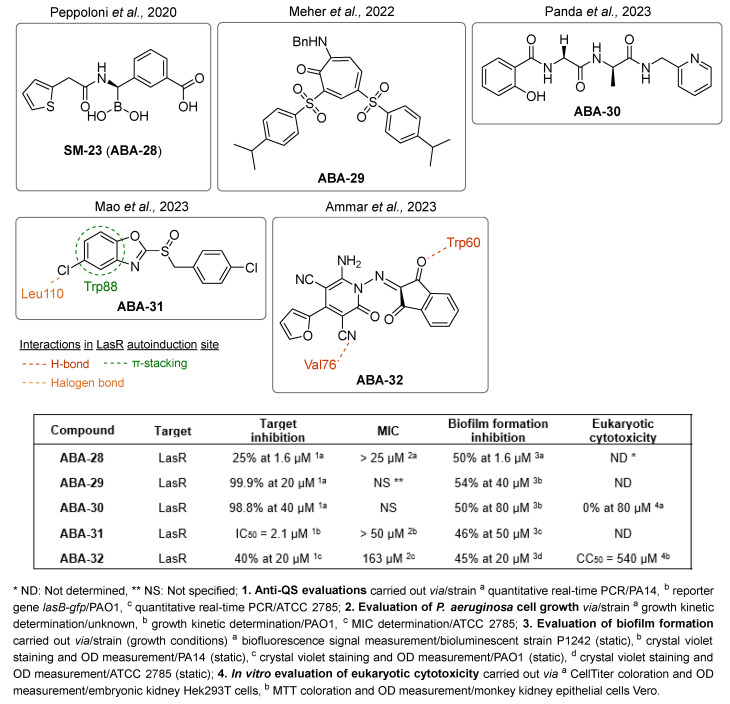
Key interactions with the target, anti-QS and anti-biofilm activities of polyaromatic QSIs targeting LasR [74,75,76,77,78].

Other QSIs were discovered via the study of relevant antimicrobial scaffolds or by exploring the anti-virulence potential of antimicrobial drugs. Several polyaromatic QSIs targeting LasR were therefore discovered. First, Peppoloni et al. studied the anti-virulence activity of the beta-lactamase inhibitor **SM-23** (**ABA-28**) (Figure 10) [74]. This boric acid derivative was able to inhibit odDHL and BHL secretion by 77 and 31%, respectively, as well as some virulence factors such as pyoverdine, elastase and pyocyanin. **SM-23** decreased 50% of both biofilm formation and maturation at a concentration as low as 1.6 µM. Interestingly, **SM-23** also successfully reduced the establishment of biofilm onto endotracheal tubes in an in vitro model mimicking clinical settings. Nonetheless, this LasR inhibitor was not described further since this study was conducted in 2020. Some QSIs have been brought out by rational design based on the literature data. For instance, Meher et al. reported in 2022 novel troponyl-sulfone derivatives inspired by (i) natural troponoid antimicrobial products such as thuyaplicin associated with (ii) the sulfone of the beta-lactamase inhibitor sulfabactam [75]. Therefore, the compound **ABA-29** exhibited potent anti-LasR and anti-LasI properties (almost 100% inhibition at 20 µM), which are correlated with interesting anti-biofilm activity (54% inhibition of biofilm formation at 40 µM). No eukaryotic cytotoxicity was detected. A treatment with **ABA-29** on an epithelial kidney cell line infected with *P. aeruginosa* enabled the survival of more than 80% of the cells (vs. 37% in the absence of treatment). The same team then developed new *N*-salicyl-AA_n_-picolylamine pseudo-peptides inspired by the encouraging QS inhibitory properties of salicylic acid derivatives [76]. Interestingly, unlike most of its analogs, the hit compound *N*-salicyl-Gly-Ala-picolylamine **ABA-30** did not affect the bacterial membrane, and decreased biofilm formation by 50% at 80 µM through potent inhibition of LasR (almost 100% at 40 µM). In 2023, Mao et al. designed benzoxazolo-sulfoxides as QSIs, taking into account the encouraging literature data [77]. Among the series, the compound **ABA-31** showed the best anti-biofilm activity with 46% inhibition of biofilm formation at 50 µM through potent LasR inhibition (IC_50_ = 2.1 µM). According to the molecular docking study, its 4-chloro-benzyl moiety occupies the same cavity as the odDHL alkyl chain in the LasR binding site, while the 5-chloro-benzoxazole establishes a π-stacking interaction with Trp88 and a halogen-bond with Leu110. These findings corroborate their SAR study that revealed the necessity of those groups for anti-biofilm activity. Finally, Ammar et al. described a series of 2-oxo-pyridines as novel anti-biofilm molecules targeting LasR [78]. The most active product, **ABA-32**, inhibits biofilm formation by 45% at 20 µM without any eukaryotic cytotoxicity. The establishment of two H-bonds with Trp60 and Val76 would greatly stabilize the complex with LasR.

(2) QSIs targeting PqsR

**Figure 11:**
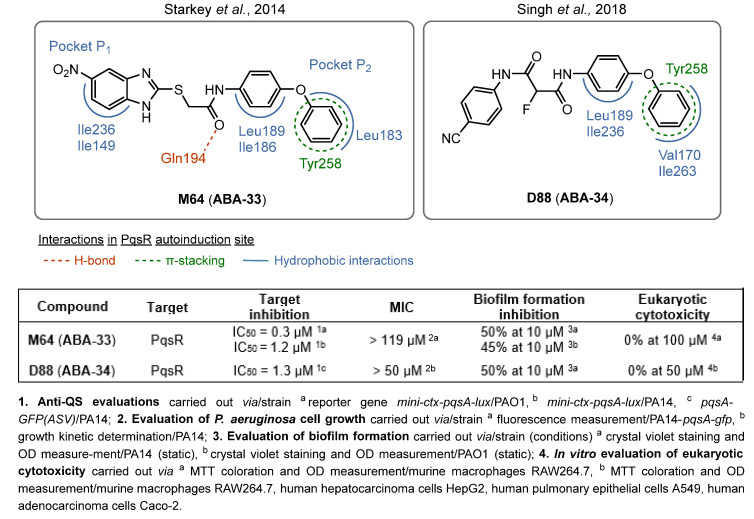
Key interactions with the target, anti-QS and anti-biofilm activities of **M64** and **D88** as biaromatic PqsR inhibitors [79,80,81].

The benzamide–benzimidazole hybrid **M64** (**ABA-33**) identified by Starkey et al. in 2014 is one of the first described PqsR inhibitors and still one of the best to date (IC_50_ = 0.3 and 1.2 µM for PAO1 and PA14, respectively) [78,79,80]. Therefore, **M64** is a very potent inhibitor of pyocyanin secretion (IC_50_ = 0.3 µM in PA14 strain) and of biofilm production. This QSI notably reduces 50 and 45% of biofilm formation in PA14 and PAO1 strains at 10 µM, respectively (Figure 11). Against antibiotolerant preformed biofilms, it acts in vitro in synergy with meropenem and tobramycin to increase their efficacy. Moreover, **M64** did not show any cytotoxicity on RAW264.7 macrophage cells below 100 µM. In a murine model of burn and lung PA14 infections, this compound was able to significantly reduce bacterial virulence and improve survival rates, potentializing the immune response. In combination with ciprofloxacin, **M64** improved the ATB efficacy in a subtherapeutical concentration for a shorter treatment duration and prevented the recrudescence of the infection. In 2018, the team explored its anti-QS mechanism of action by co-crystallizing its complex with PqsR (PDB-6B8A) [81]. Interestingly, the QSI occupies the same hydrophobic pockets P_1_ and P_2_ in the autoinduction domain as the native ligand PQS. However, the binding of **M64** to PqsR results in a conformation change, allowing the molecule to establish key hydrophobic interactions with the Leu183 and Ile186 residues. Its central sulfur atom allows it to take a suitable geometry and fit exactly in the PqsR autoinduction site. In addition, the carbonyl group of **M64** forms an H-bond with Gln194 and its phenyl ring establishes a π-stacking with Tyr258, both of which are not involved in the interaction between PqsR and its native ligand. **M64** acts therefore as a competitive inhibitor of PqsR thanks to a new potent binding mode. Although **M64** has remarkable anti-biofilm efficacy, its biopharmaceutical profile needs to be improved. Indeed, the presence of a potentially genotoxic nitro group and its poor aqueous solubility disfavored its further development. Pharmacomodulations led to the discovery of the derivative **D88** (**ABA-34**) based on an *N*,*N*-biaryl malonamide scaffold, which presents very potent anti-PqsR activity (IC_50_ = 1.31 µM) [82]. **D88** inhibits 50% of biofilm formation in the PA14 strain at 10 µM, reduces pyocyanin secretion with an IC_50_ of 0.53 µM and possesses a more favorable ADMET (Absorption, Distribution, Metabolism, Excretion, Toxicity) profile than its analog **M64** (aqueous solubility of 490 µM in PBS, no cytotoxicity on several eukaryotic cell lines). Its in vivo activity was assessed in monotherapy on a murine burn PA14 infection model. Interestingly, **D88** could inhibit the bacterial dissemination towards intestinal tissues. It seems that **D88** can diminish the morphological alteration of intestinal cells mediated by PA14 infection. This finding represents a significant improvement in the search for treatments against hard-to-treat *P. aeruginosa* infections associated with gut-deprived sepsis. Therefore, **D88** exhibits anti-QS and anti-virulence properties as potent as **M64**. A molecular docking study indicated a similar binding of **D88** in the autoinduction site of PqsR as **M64**. Notably, **D88** establishes hydrophobic interactions with Leu189 and Ile236 as well as a π-stacking with Tyr258.

QSIs are highly promising ABAs. Moreover, they often possess broad anti-virulence properties including an inhibition of pyocyanin, rhamnolipid or elastase production, or a reduction in bacterial motility. At first, most research works focused on the development of *pan*-QSI targeting LasR. Hence, some non-analogs of odDHL such as **ABA-21** and **ABA-28** appear very potent, with 50% inhibition of biofilm formation from 10 µM and at 1.6 µM, respectively. Yet, the *pqs* system has been more and more studied due to its pseudomonal specificity, whereas the *las* and *rhl* circuits are AHL-dependent communication networks that can be found in many bacterial species. Among the most promising ABAs, one of the first relevant PqsR inhibitors **M64** (**ABA-33**) and its recent derivative **D88** (**AAB-34**) showed powerful anti-QS and anti-virulence efficacy thanks to a competitive inhibition of their target. The improvement of **D88**’s biopharmaceutical profile makes it an even more interesting QSI. The AQ analogs **ABA-12** and **ABA-14**, bearing a pyridinone or pyridine core, also display highly promising anti-biofilm efficacies with IC_50_ equal to 4.5 and below 0.5 µM, respectively. Therefore, the understanding of key structural features for ligand–target interaction as well as the development of new in silico tools for ADMET predictions enable the rational design of novel potent molecules. A wise use of such knowledge may still pave the way for further promising discoveries.

#### 3.1.3. Lectin Inhibitors

The soluble lectins LecA and LecB are present on the outer membrane of the bacteria to enable adherence to host tissues, a critical step in the infection initiation process. They also ensure the intercellular adhesion inside the biofilms and strengthen their building. Therefore, lectins have triggered the interest of medicinal chemists as new therapeutic targets against *P. aeruginosa* infections. These ABAs inhibit LecA and LecB fixation to D-galactose or L-fucose monosaccharides. By analogy, the structure of the inactivating ligands is based on the same patterns [37].

**Figure 12:**
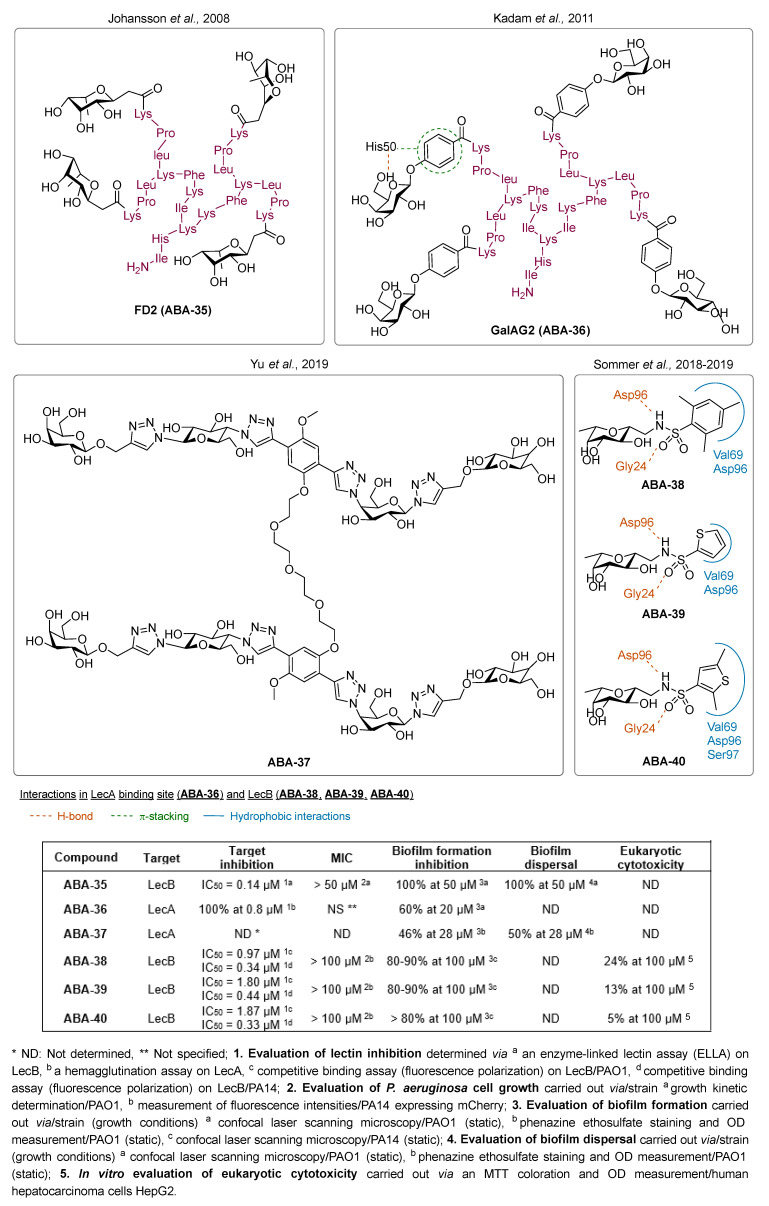
Key interactions with the target, anti-lectin and anti-biofilm activities of lectin A and B inhibitors (the peptide backbone appears in purple) [82,83,84,85,86].

One major drawback for the conception of the first monovalent lectin inhibitors is the weakness of the lectin/ligand bond. On the contrary, it has been revealed that the binding of multivalent carbohydrate derivatives to lectins induces a cluster effect that strongly inactivates the protein. Johansson et al. designed a series of synthetic multivalent fucose-based ligands with a high affinity for LecB [83]. These glycodendrimers were assembled on a peptide backbone using solid-phase synthesis methods, which allowed amino acid modulations to reach greater specificity or refine their physicochemical properties. Of the two peptide dendrimers’ developed libraries, nine compounds inhibited LecB in an enzyme-linked lectin assay (ELLA) (IC_50_ = 0.11 to 0.75 µM) with a 100-fold activity increase compared to L-fucose (IC_50_ = 11 µM). Remarkably, the compound **FD2** (**ABA-35**) was able to completely inhibit biofilm formation at 50 µM (Figure 12). At this concentration, **FD2** also induced a complete elimination of mature biofilms established on an abiotic surface, including those of three cystic fibrosis-derived clinical strains. This makes it a very interesting agent for the treatment of *P. aeruginosa* biofilm-related infections. A model of the **FD2**-LecB complex showed that the molecule was in fact too small to be multivalent and to interact with two binding sites. However, the compound forms H-bonds and hydrophobic interactions with residues outside the binding sites, which explains its strong affinity for LecB. Kadam et al. applied the same strategy to the design of multivalent ligands targeting the galactose-specific lectin LecA [84]. The peptide dendrimer portion of **FD2** was thus attached to a carboxyphenyl β-galactoside moiety to obtain the derivative **GalAG2** (**ABA-36**), which shows an 875-fold increase in binding to LecA compared to D-galactose. **GalAG2** inhibits 60% of biofilm formation at 20 µM. Interestingly, its monovalent analogs were revealed to be inactive, demonstrating that multivalency was crucial to anti-biofilm efficacy. Yu et al. also developed the tetravalent anti-biofilm compound **ABA-37** to target LecA [85]. In this molecule, a flexible PEG spacer allows the cross-linking between two central phenyl groups. **ABA-37** inhibits 46% of biofilm formation at 28 µM. At the same concentration, the molecule also successfully induces a 50% dispersion of a preformed biofilm. Thus, this kind of structure is likely to be interesting for the development of new anti-biofilm agents. Still, optimization is required to improve their binding affinity to lectins. However, these large multivalent dendrimers possess a high molecular weight. In order to avoid undesirable cross-links inside the biofilm and to enhance their chances to reach a drug-like molecule, Sommer et al. decided to focus on monovalent low molecular weight inhibitors of LecB and developed aryl-sulfonamide glycomimetics [86]. The best anti-biofilm results were obtained with the two compounds **ABA-38** and **ABA-39**, which inhibited 80-90% of biofilm formation at 100 µM. Moreover, both molecules showed a good affinity towards LecB, with IC_50_ of 0.34 and 0.44 µM for **ABA-38** and **ABA-39**, respectively. Their orientation in the LecB binding site appears comparable to that of D-mannose and enables the formation of H-bonds with Asp96 and Gly24 while the aromatic ring interacts with the side chains of Val69 and Asp96. On top of that, other interactions such as lipophilic contacts between the *c*-glycosidic methyl group and Thr45 and Ala23 are a key feature for further enhancement of the affinity towards LecB. Finally, **ABA-38** and **ABA-39** revealed no toxicity as well as good metabolic stability and oral bioavailability in vivo. These glycomimetics could thus be used in further assays as monotherapy and in combination with antibiotics against biofilm-associated infections. These two compounds were used as hits for the development of the 2,5-dimethyl thiophene derivative **ABA-40** [87]. This ABA shows slightly increased potency to quench LecB thanks to additional lipophilic interactions with the side chains of Val69, Asp96 and Ser97. Remarkably good ADMET properties of this compound combined with potent anti-biofilm activity (>80% biofilm mass reduction at 100 µM) made it the final lead in the aryl-sulfonamide glycomimetic series.

The inhibition of lectins has proved to efficiently disturb the establishment of biofilms. Multivalent lectin inhibitors like **FD2** (**ABA-35**) are effective at reducing biofilm formation and inducing its dispersal. However, those molecules are hard to synthesize, have high molecular weights and various disadvantages including undesirable bacterial aggregation promotion and harmful immune system interferences. Nevertheless, monovalent glycomimetics displaying better ADMET properties showed potent inhibitory activity towards LecB. Those interesting results could be a first step towards the development of new drug-like lectin inhibitors.

#### 3.1.4. Antimicrobial Peptides

The antimicrobial peptides (AMPs) are small amphiphilic, cationic molecules usually composed of 10–50 residues. They are naturally produced by mammals, amphibians and fishes, micro-organisms and insects and act as a first line of defense for the innate immune system. These peptides are known to induce bacterial death by membranolytic and/or intracellular mechanisms quenching the cell cycle progression. They have triggered the interest of medicinal chemists due to their fast bactericidal mode of action and the absence of resistance appearance after repeated exposition [88]. Interestingly, some AMPs also possess anti-biofilm activities. This can either be due to (i) the eradication of sessile bacteria inside the biofilms, associated with an increased ability to penetrate through the matrix in comparison with the conventional antibiotics, or to (ii) anti-biofilm activity at a sub-MIC concentration like an AVA, hindering the biofilm cycle without any effect on bacterial growth. In this review, we are interested in those AMPs able to inhibit biofilm formation or to stimulate its dispersal.

**Figure 13:**
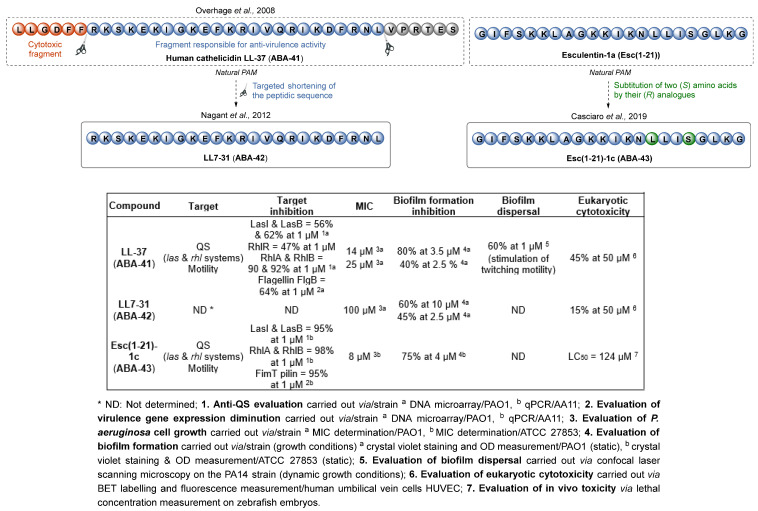
Rational design and anti-biofilm activities of AMPs derived from natural AMPs [87,88,89,90,91].

Most synthetic AMPs of interest are derived from natural AMPs. The human cathelicidin **LL-37** (**ABA-41**) has originated several analogs due to its potent anti-biofilm activity towards *P. aeruginosa*, with an 80% reduction in biofilm formation at 3.5 µM (Figure 13). Overhage et al. [89] highlighted the deleterious effect of the peptide towards initial attachment of the cells to a surface and its ability to inactivate the *las* and *rhl* QS systems, as well as its promoting effect on twitching motility, which helps dispersing the biofilm (60% dispersal at 1 µM). The design of synthetic derivatives aims at diminishing the peptide’s length, its sensitivity to proteases and saline media as well as its moderate cytotoxicity towards eukaryotic cells (45% permeabilization of HUVEC epithelial cell line at 50 µM). Nagant et al. thus developed a library of truncated fragments derived from **LL-37**, conserving the active part of the peptide only [90]. A screening brought out the AMP **LL7-31** (**ABA-42**), which is less cytotoxic (15% permeabilization of HUVEC cells at 50 µM) and slightly less efficient than **LL-37** (60% inhibition of biofilm formation at 10 µM). The facilitated synthesis of smaller peptides seems to favor better metabolic stability and lower eukaryotic cytotoxicity. Similarly, from the peptide esculentin-1a (**Esc-(1-21)**), unstable and toxic in vivo (LD_50_ = 12 µM in a zebrafish model), Casciaro et al. constructed the diastereoisomer **Esc(1-21)-1c** (**ABA-43**) by replacing Leu14 and Ser17 with their D-enantiomers ((*R*) configuration) [91,92]. This modification destabilizes the helical folding and thus increases the peptide’s stability towards proteases. The anti-biofilm activity was conserved with 75% inhibition of biofilm formation at 4 µM, with a diminished toxicity (LD_50_ = 124 µM in a zebrafish model). The peptide also reduces pyoverdine and rhamnolipid secretion (by 60 and 20%, respectively, at 1 µM), as well as bacterial swimming motility (55% inhibition at 1 µM).

**Figure 14:**
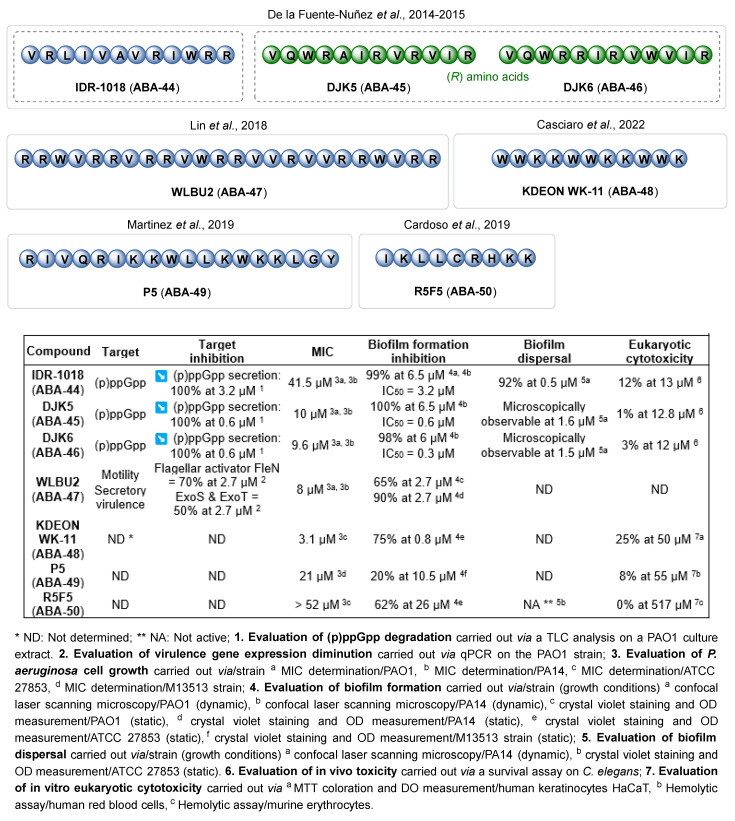
Anti-biofilm activities of AMPs developed de novo by rational design and/or by computer-assisted conception [93,94,96,97,98,101].

The synthesis of AMPs based on natural peptides has led to some highly interesting compounds, with more efficacy and less toxicity. However, new knowledge now enables a de novo rational design, with or without the use of informatic tools. This approach favors the optimization of the helical and amphipathic structure, charge and hydrophobic densities of the new AMPs. De la Fuente-Nuñez et al. have studied several small anti-biofilm peptides that are efficient against *P. aeruginosa* [93]. They notably identified the compound **IDR-1018** (**ABA-44**) from a screening of a small cationic peptide library (Figure 14). This AMP has a broad-spectrum action favoring the degradation of the 5′ (tri)diphosphate 3′-diphosphate guanosine (p)ppGpp, a secondary messenger implicated in the bacterial response to environmental stress and in the stimulation of biofilm production pathways. The **IDR-1018** AMP inhibits 99% of biofilm formation at 6.5 µM (IC_50_ = 3.2 µM) and induces 92% dispersal of a preformed biofilm at 0.5 µM. To address in vivo instability and cytotoxicity issues observed with most of the AMPs, the team then described two small peptides, **DJK5** (**ABA-45**) and **DJK6** (**ABA-46**), composed with D-aa ((*R*) configuration) [94]. **DJK5** and **DJK6** inhibit biofilm formation with IC_50_ of 0.6 and 0.3 µM, respectively, by activating (p)ppGpp degradation. Moreover, **DJK6** shows a synergistic action with a non-bactericidal concentration of ciprofloxacin (0.12 µM) to completely inhibit biofilm formation at 0.6 µM. The ATB efficacy is also restored against a mature biofilm in association with this AMP stimulating its dispersion. Contrary to **IDR-1018**, **DJK6** could protect *C. elegans* larvae from *P. aeruginosa* infection (90% survival vs. 1%, 48 h post-infection). Therefore, the development of D-enantiomeric peptides enables a cytotoxicity diminution and an increased resistance to proteases without impairing the anti-biofilm activity. Then, the importance of a few redundant amino acids for AMP anti-biofilm properties has been pointed out. Therefore, the **WLBU2 **(**ABA-47**) sequence is only composed of arginine, tryptophane and valine residues [95,96]. **WLBU2** appears highly attractive for its anti-biofilm efficacy (65% and 90% inhibition of biofilm formation at 2.7 µM against PAO1 and PA14 strains, respectively) due to a decreased motility and a reduction in secretory virulence. The smaller peptide **KDEON WK-11** (**ABA-48**) is composed of a repeated sequence of eleven tryptophan and lysine residues, arranged in a way that promotes the amphipathic equilibrium of the alpha helix [97]. Interestingly, **KDEON WK-11** does not induce significant cytotoxicity towards eukaryotic cells and exhibits one of the most interesting anti-biofilm properties in the literature, with 75% biofilm formation inhibition at 0.8 µM. In 2019, Martinez et al. described peptide **P5** (**ABA-49**), designed de novo thanks to both rational conception and computer-assisted design. Using predicting tools such as ClustalX, HeliQuest and HydroMCalc, they could tune the cationicity, hydrophobicity and helicity of their peptides at will. The **P5** compound inhibited 20% of biofilm formation at 10.5 µM [98,99]. Cardoso et al. conceived their AMP **R5F5** (**ABA-50**) with the help of the JOKER algorithm [100]. Reported by Porto et al., this tool helps inserting relevant patterns to create novel, more active AMPs [101]. **R5F5** decreases biofilm production by 62% at 26 µM without any hemolytic cytotoxicity and appears as a hit compound for further optimizations.

The development of anti-biofilm AMPs that do not hinder bacterial growth appears as a promising strategy against *P. aeruginosa* infections. Their mode of action can either implicate (i) an inhibition of biofilm formation, decreasing the bacterial motility or quenching the bacterial communication or metabolic regulation pathways, or (ii) a stimulation of the biofilm dispersal. The rational design of small cationic peptides such as the D-AMP **DJK6** and the redundant peptide **KDEON WK-11** revealed their interesting anti-biofilm efficacy at low concentrations, with lower eukaryotic cytotoxicity, better in vivo stability and lower production costs. It is noteworthy that these two peptides can also eradicate preformed biofilms at bactericidal concentrations (100% eradication at 10 µM for **DJK6** and 50% at 6 µM for **KDEON WK-11**). Finally, their use at sub-MIC concentrations offers synergistic activity with some ATBs that seem particularly interesting in the struggle against antibioresistant *P. aeruginosa* infections while circumventing the major drawback of AMPs’ inherent cytotoxicity.

#### 3.1.5. Conventional Antibiotic Repurposing and Anti-Biofilm Nanocarriers

**Figure 15:**
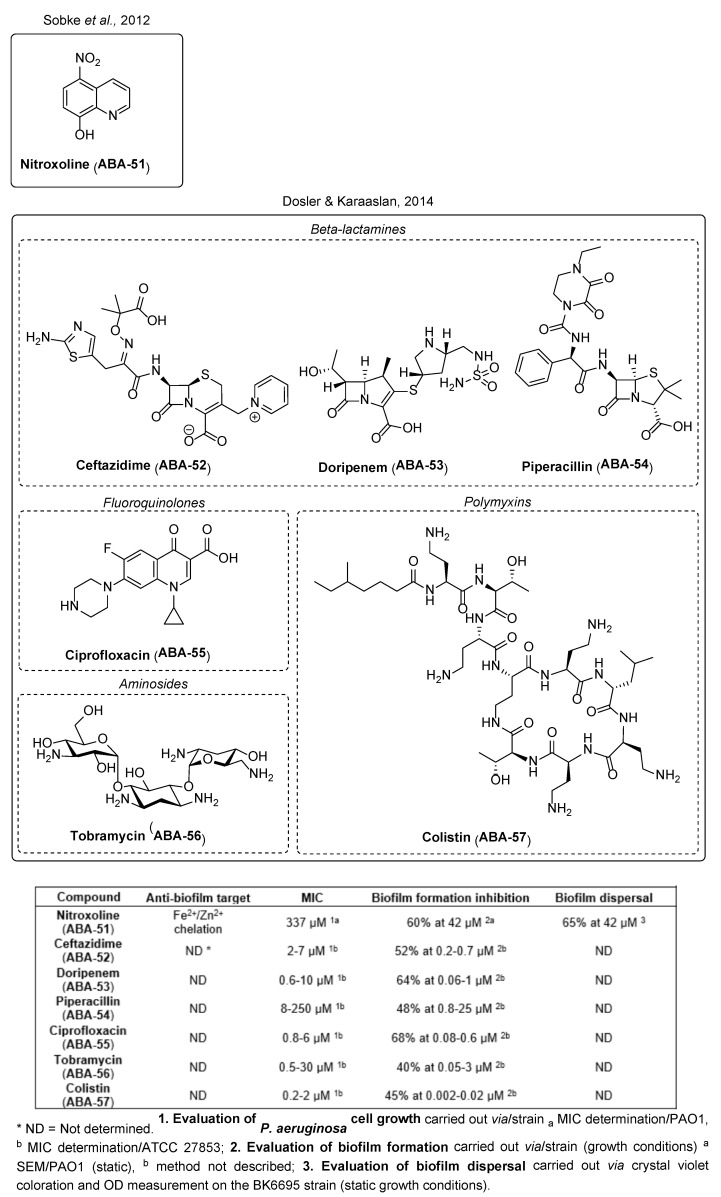
Anti-biofilm activities of conventional antibiotics [102,103].

Like antimicrobial peptides, some conventional antibiotics also show anti-biofilm efficacy at sub-MIC concentrations. Although their anti-virulence targets mostly remain uncertain, their action against the biofilm formation or in favor of its dispersion seems promising. For instance, 5-nitro-8-hydroxyquinoline (nitroxoline, **ABA-51**) inhibits 60% of biofilm formation at 42 µM (8 µg/mL, ¼ MIC) by complexing essential ions for the bacterial metabolism such as iron or zinc (Figure 15) [102]. Moreover, nitroxoline stimulates the dispersal of 65% of a preformed biofilm, according to the stimulation of twitching motility. In 2014, a study conducted by Dosler and Karaaslan also highlighted the anti-biofilm potential of six other conventional ATBs against *P. aeruginosa* [103]. Ceftazidime (**ABA-52**), doripenem (**ABA-53**), piperacillin (**ABA-54**), ciprofloxacin (**ABA-55**), tobramycin (**ABA-56**) and colistin (**ABA-57**) inhibited the formation of biofilm by 40 to 68% at 1/10 MIC, i.e., in the range of a tenth of a micromolar. Those ATBs decrease between 22 and 62% bacterial attachment during the four first hours of biofilm establishment, at 1/10 MIC. However, they have no effect on mature biofilms. Colistin appears the most interesting conventional ATB for use as an ABA by reducing 45% of biofilm formation at 2 nM, i.e., 1/100 MIC. It is yet a last-resort ATB because of its high nephrotoxicity.

**Figure 16:**
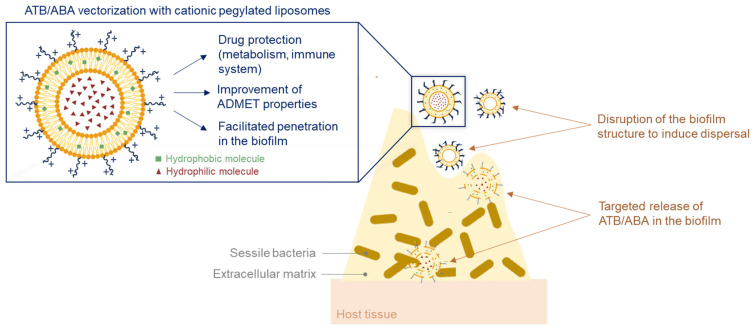
Targeted transport of ATB/ABA with cationic pegylated liposomes.

Nanocarriers are another novel promising anti-virulence approach for biofilm inhibition. In fact, the last few years have seen a growing interest in the development of liposomal formulations able to restore ATBs’ efficacy by improving their penetration inside the extracellular matrix. The encapsulation of molecules or their attachment to the nanovector surface protects them from metabolization, improves their bioavailability, reduces their toxicity and increases their efficacy by ensuring a targeted delivery (Figure 16) [104]. Moreover, it has been shown that small spherical nanocarriers can diffuse through the extracellular matrix and could therefore deliver ATBs. Liposomes, biomimetics of cell membranes, offer a transport for both hydrophilic (inside the core) and hydrophobic (in the lipidic bilayer) molecules. The liposomal formulas are easily modifiable, enabling the refinement of their physicochemical properties such as their size, shape, charge, hydrophilicity or lipophilicity to optimize the drug’s transport towards the action site. Their surface can be functionalized with polyethylene glycols (PEGs), which improve their biodistribution by protecting them from macrophages. The surface charge and the pegylation of liposomes also influence their interactions with biofilms. Hence, Ibaraki et al. have described that cationic pegylated liposomes are more efficient against biofilms than their anionic counterparts as they possess stronger electrostatic affinity with the negatively charged biofilm surface and a better matrix permeability [105]. Interestingly, a treatment with empty liposomes can also destabilize the biofilm and trigger its dispersal.

### 3.2. Eradication of Biofilm-Associated Infections: Combinatorial Approach AVA/ATB

The use of anti-biofilm agents could prevent biofilm formation and/or help dispersing existing biofilms. Combining an anti-biofilm agent with a conventional antibiotic could thus restore or even potentiate its efficacy against chronic and hard-to-treat infections.

**ABA-4**, developed by Andersen et al. as a c-di-GMP pathway inhibitor, shows very interesting efficacy in synergy with ciprofloxacin or tobramycin by promoting biofilm disruption, thus enabling the killing of dispersed cells by both ATBs in vitro [46]. While ciprofloxacin and tobramycin only eradicated 33 and 8% of bacteria, respectively, the pre-treatment with 100 µM of **ABA-4** led to the elimination of 84 and 57% of bacteria, respectively (Table 1). This antimicrobial activity enhancement is also observed in vivo on a murine model of PAO1 biofilm infection for which the administration of **ABA-4** prior to the ATB treatment improved bacterial clearance. This molecule could be a lead for the development of drugs to be used in combination with ATBs for the treatment of cystic fibrosis lung, wound or urinary tract infections as well as VAP. Another compound, **ABA-58**, described by Thomann et al. also restored ciprofloxacin’s efficacy against *P. aeruginosa* biofilms [106]. This molecule is not a very potent ABA but diminishes the levels of eDNA, polysaccharides and proteins which constitute the biofilm matrix. Therefore, while the single use of ciprofloxacin eradicated 28% of PA14 bacterial cells in an in vitro biofilm assay, its combination with 50 µM of **ABA-58** significantly increased its efficacy, up to 62%. In the same way, the benzamide–benzimidazole hybrid **M64** (**ABA-36**) has an in vitro synergistic activity from 10 µM with meropenem or tobramycin against *P. aeruginosa* PA14 biofilms. Together, they decrease cell viability by 34% and 66%, respectively, vs. 3 and 24%, respectively, in ATB monotherapy [79,80]. The authors demonstrated that **M64** potentiates the ability of both ATBs to disrupt biofilms in pre-treatment or concomitant treatment. Then, the quinazolinone **ABA-59** developed by Murray et al. revealed an original dual-efficacy with tobramycin against mixed-species biofilms of *P. aeruginosa* and *S. aureus* [107]. Indeed, the single use of **ABA-59** thus unusually eradicated *S. aureus* but not *P. aeruginosa*, whereas a monotherapy with tobramycin only killed the pseudomonal species (60% eradication at 214 µM). However, the combination of both **ABA-59** and tobramycin resulted in 95% eradication of both species in the biofilm. In fact, the ABA notably inactivates the production by *P. aeruginosa* of the QS secondary metabolite HQNO, which is able to decrease the sensitivity of *S. aureus* to tetracyclines. The ability of **ABA-59** to potentialize the efficacy of tobramycin against these mixed-species biofilms could be of great use for the treatment of cystic fibrosis infections in which both bacteria often co-exist. Soukarieh et al. [62] also evaluated the synergistic anti-biofilm potential of **ABA-18** with tobramycin. When cultured in the presence of **ABA-18**, the biomass was diminished without any effect on cell viability and a subsequent treatment with tobramycin improved biofilm eradication compared to a tobramycin monotherapy (95% inhibition of cell viability in dual therapy vs. 75% in ATB monotherapy). Finally, the compound **GM-50** (**ABA-60**) described by Bernabè et al. potentiated in combination the capacity of aztreonam to disrupt biofilms (60% biofilm dispersal vs. 40% in monotherapy) [108]. Interestingly, the molecular docking study indicated an affinity of **GM-50** towards RhlR.

Some AMPs with interesting anti-biofilm activities have also been studied for their ability to potentiate ATBs’ efficacy against biofilm-related infections (Table 2). Peptide **IDR-1018** (**ABA-44**), designed by De la Fuente Nuñez, is a broad-spectrum anti-biofilm AMP which triggers the degradation of the (p)ppGpp signal, inhibiting biofilm formation and leading to the dispersion of mature biofilms [109]. Against a mature biofilm, the combination of **IDR-1018** with ciprofloxacine enables a total clearance of sessile bacteria. The D-AMP **DJK6** (**ABA-46**) also showed interesting synergistic activity with ciprofloxacin, with a complete prevention of biofilm formation associated with a complete eradication of a preformed biofilm [94].

## 4. Conclusions

Bacterial biofilms are an aggravating factor in *P. aeruginosa* infections as they provide optimal conditions for (i) bacterial cell growth, (ii) protection against the immune system and antibiotics and (iii) the acquisition of resistance phenotypes, which make infections very hard to treat. The understanding of biofilm communities and underlying mechanisms is a first step towards the development of anti-biofilm strategies. It brings out relevant targets for drug development. In fact, the discovery of anti-biofilm agents could help to fight infections while reducing the emergence of resistances and restoring the efficiency of conventional ATBs when used in dual therapy. The rational design of molecules offers easy access to efficient compounds with improved physiochemical properties and reduced cytotoxicity. In particular, some QSIs targeting the *pqs* system appear very interesting. The AQ analogues **ABA-12** and **ABA-14** possess very potent anti-biofilm activity (IC_50_ = 4.5 µM for **ABA-12** and IC_50_ ≤ 0.5 µM for **ABA-14**). **M64** and its derivative **D88**, biaromatic competitive PqsR inhibitors, can reduce biofilm formation and show synergistic activity with conventional ATBs against preformed biofilm. Some AMPs such as the D-AMP **DJK6** and the repetitive peptide **KDEON WK-11** have also revealed very promising anti-biofilm properties at low concentrations (IC_50_ = 0.3 µM for **DJK6** and 75% inhibition at 0.8 µM for **KDEON WK-11**). It should be noted that there is as yet no mutual agreement on biofilm infection models, which limits effective comparisons between potent anti-biofilm agents. Finally, the emergence of new biotechnologies such as nanocarriers could solve problems of bioavailability or toxicity. Progress in research into anti-biofilm agents appears very encouraging for future therapies.

## Figures and Tables

**Table 1:** Biological activities of ATB and AVA combinations.

Combination				Biofilm Eradication		Biofilm Dispersal		Ref
ATB		AVA		ATB	ATB + AVA	ATB	ATB + AVA	
Structure	Posology	Structure	Posology					
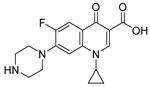 **Ciprofloxacin**	1.5 µM	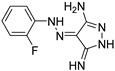 **ABA-4**	100 µM	33% ^1a^	84% ^1a^	ND *		[46]
	1 µM	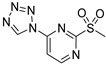 **ABA-58**	50 µM	28% ^1b^	62% ^1b^	ND		[106]
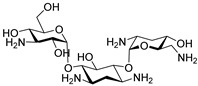 **Tobramycin**	64 µM	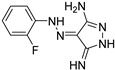 **ABA-4**	100 µM	8% ^1a^	57% ^1a^	ND		[46]
	21 µM	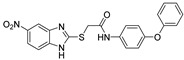 **ABA-36**	10 µM	24% ^1c^	66% ^1c^	ND		[80]
	214 µM	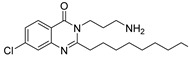 **ABA-59**	100 µM	60% ^1d^	95% ^1d^	ND		[107]
	214 µM	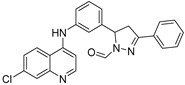 **ABA-18**	8 µM	75% ^1e^	95% ^1e^	ND		[62]
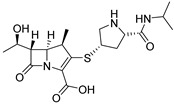 **Meropenem**	26 µM	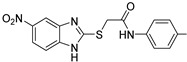 **ABA-36**	10 µM	3% ^1c^	34% ^1c^	ND		[80]
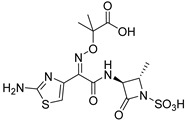 **Aztreonam**	9 µM	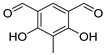 **ABA-60**	100 µM	ND		40% ^2^	60% ^2^	[108]

* ND: Not determined; **1. Evaluation of biofilm eradication** carried out via ^a^ CFU/mL determination on a 24 h pre-grown biofilm treated with AVA for 2 h then with ATB for 8 h, ^b^ CFU/mL determination on a 24 h PA14 pre-grown biofilm in presence of AVA then treated with ATB for 24 h, ^c^ CFU/mL determination on a 48 h PA14 pre-grown biofilm in presence of AVA then treated with ATB for 24 h, ^d^ biofilm biomass quantification using confocal microscopy on a 48 h PAO1 pre-grown biofilm in presence of AVA then treated with ATB for 4 h, ^e^ biofilm biomass quantification using confocal microscopy on a 16 h PAO1 pre-grown biofilm in presence of AVA then treated with ATB for 4 h; **2. Evaluation of biofilm dispersal** carried out by measurement of relative fluorescence units (RFUs) on a 24 h PAO1 pre-grown biofilm treated with AVA and ATB for 16 h.

**Table 2:** Biological activities of ciprofloxacin and anti-biofilm AMP combinations.

Combination				Biofilm Formation Inhibition		Biofilm Dispersal		Biofilm Eradication		Ref
ATB		AMP		ATB	ATB + AMP	ATB	ATB + AMP	ATB	ATB + AMP	
Structure	Posology	Structure	Posology							
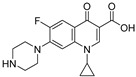 **Ciprofloxacin**	0.12 µM	**ABA-44 (1018)**	0.52 µM	ND *	ND	ND	ND	NS **	90% ^3a^	[109]
		**ABA-45 (DJK5)**	0.064 µM	ND	100% ^1a^	ND ^2a^	Weak ^2a^	ND ^3b^	100% ^3b^	[94]
		**ABA-46 (DJK6)**	0.6 µM	ND	100% ^1a^	ND ^2a^	100% ^2a^	ND ^3b^	100% ^3b^	

* ND: Not determined; ** NS: Not significant; **1. Evaluation of biofilm formation** carried out via/strain ^a^ confocal fluorescence microscopy/PA14 **2. Evaluation of biofilm dispersal** carried out via ^a^ confocal microscopy on a 72 h grown PA14 biofilm in presence of AMP and ATB; **3. Evaluation of biofilm eradication** carried out via ^a^ CFU/mL determination on a 48 h PA14 pre-grown biofilm treated with AMP and ATB for 3 h, ^b^ confocal microscopy on a 72 h grown PA14 biofilm in presence of AMP and ATB.

## Abbreviations

The following abbreviations are used in this review:

ABA	anti-biofilm agent
ADMET	administration, distribution, metabolism, excretion, toxicity
AI	autoinducer
AMP	antimicrobial peptide
ATB	antibiotic
CC50	cytotoxic concentration 50%
CF	cystic fibrosis
CFU	colony forming unit
HGT	horizontal gene transfer
IC50	inhibitory concentration 50%
MIC	minimal inhibitory concentration
OD	optical density
PDB	protein data bank
PDE	phosphodiesterase
QS	quorum sensing
QSI	quorum sensing inhibitor
SAR	structure–activity relationship
SCV	small colony variants
SEM	scanning electron microscopy
